# Less is more: the lack of autoinducer-2-dependent quorum sensing promotes competitive fitness of *Escherichia coli* strain 83972

**DOI:** 10.3389/fcimb.2025.1603759

**Published:** 2025-07-02

**Authors:** Marla Keizers, Krishnendu Mukherjee, Michael Berger, Ulrich Dobrindt

**Affiliations:** Institute of Hygiene, University of Münster, Münster, Germany

**Keywords:** *lsr* locus, extraintestinal pathogenic *E. coli*, fitness, stress response, competitiveness

## Abstract

Autoinducer-2 is a signaling molecule involved in quorum sensing in *Escherichia coli*. Quorum sensing enables coordinated behavior based on cell density and helps bacteria adapt to their environment. The *luxS* gene and the *lsr* locus are involved in the biosynthesis, transport, and intracellular phosphorylation of autoinducer-2. Disruption of autoinducer-2 biosynthesis or transport can reduce biofilm formation, chemotaxis, and the expression of genes relevant for the uropathogenicity of *E. coli*. Interestingly, most isolates of *E. coli* phylogroup B2, in which uropathogenic and other extraintestinal pathogenic strains are overrepresented, lack the *lsr* operon. We show that autoinducer-2-dependent quorum sensing is not fundamentally beneficial for efficient and prolonged urinary bladder colonization. We demonstrate that the *lsr*-negative asymptomatic bacteriuria isolate 83972 has a higher fitness than its *lsr*-complemented variant. Using transcriptome analyses, competitive growth assays, and comparisons of selected fitness properties, we show that restoration of the *lsr* operon in this strain background results in growth retardation, loss of competitiveness, and higher sensitivity to oxidative stress. Our results illustrate that the lack of autoinducer-2-dependent quorum sensing contributes to the well-known fitness and competitiveness of *E. coli* 83972, on which its effective use for bacterial interference in the urinary bladder relies. It is vital to delve deeper to fully understand the fitness and competitiveness of the ABU strain 83972 if we are to optimize its use in therapeutic colonization. The key is to unravel the underlying molecular mechanisms, thus ensuring the efficacy and safety of this treatment as an alternative to antibiotic therapy.

## 1 Introduction

Extraintestinal pathogenic *Escherichia coli* (*E. coli*) strains (ExPEC) are usually classified as facultative pathogens since they often colonize the gut without causing symptoms (Wiles et al., 2008; Köhler and Dobrindt, 2011). However, they harbor accessory traits that enable them to invade and colonize extraintestinal niches, eventually leading to symptomatic diseases (Vila et al., 2016; Sora et al., 2021). *E. coli* strains that have characteristic genetic markers that indicate the potential to cause extraintestinal disease, or that were directly isolated from infections outside the gastrointestinal system, are typically classified as ExPEC. ExPEC are usually divided into sepsis-causing (SEPEC), neonatal meningitis-associated (NMEC), and uropathogenic *E. coli* (UPEC), as well as strains causing systemic disease in animals such as avian pathogenic (APEC) or mammary pathogenic (MPEC) *E. coli* (Köhler and Dobrindt, 2011; Sora et al., 2021). Several virulence factors are described to promote extraintestinal pathogenicity in ExPEC, including adhesins, invasins, iron uptake systems, or toxins (Sora et al., 2021). Another bacterial trait that is involved in extraintestinal pathogenicity is quorum sensing (QS). Quorum sensing is a process in which bacteria sense and adapt to changing cell densities through signaling molecules called autoinducers (AI). Several AIs that are specific for certain bacterial strains or families have been described (Rutherford and Bassler, 2012). AI-2, on the other hand, serves as an interspecies signaling molecule that is produced and sensed by both Gram-positive and Gram-negative bacteria (Sun et al., 2004; Pereira et al., 2013). Even eukaryotic cells were proposed to participate in AI-2-mediated QS since it was found that *Saccharomyces cerevisiae* secretes molecules that mimic AI-2, as well as the intestinal epithelial cell (IEC) line Caco-2 in response to bacteria or a tight-junction disruption (Ismail et al., 2016; Valastyan et al., 2021). Moreover, the inflammatory interleukin IL-8 was found to be upregulated in response to external AI-2 in the IEC line HCT-8 (Zargar et al., 2015).

**Figure 1:**
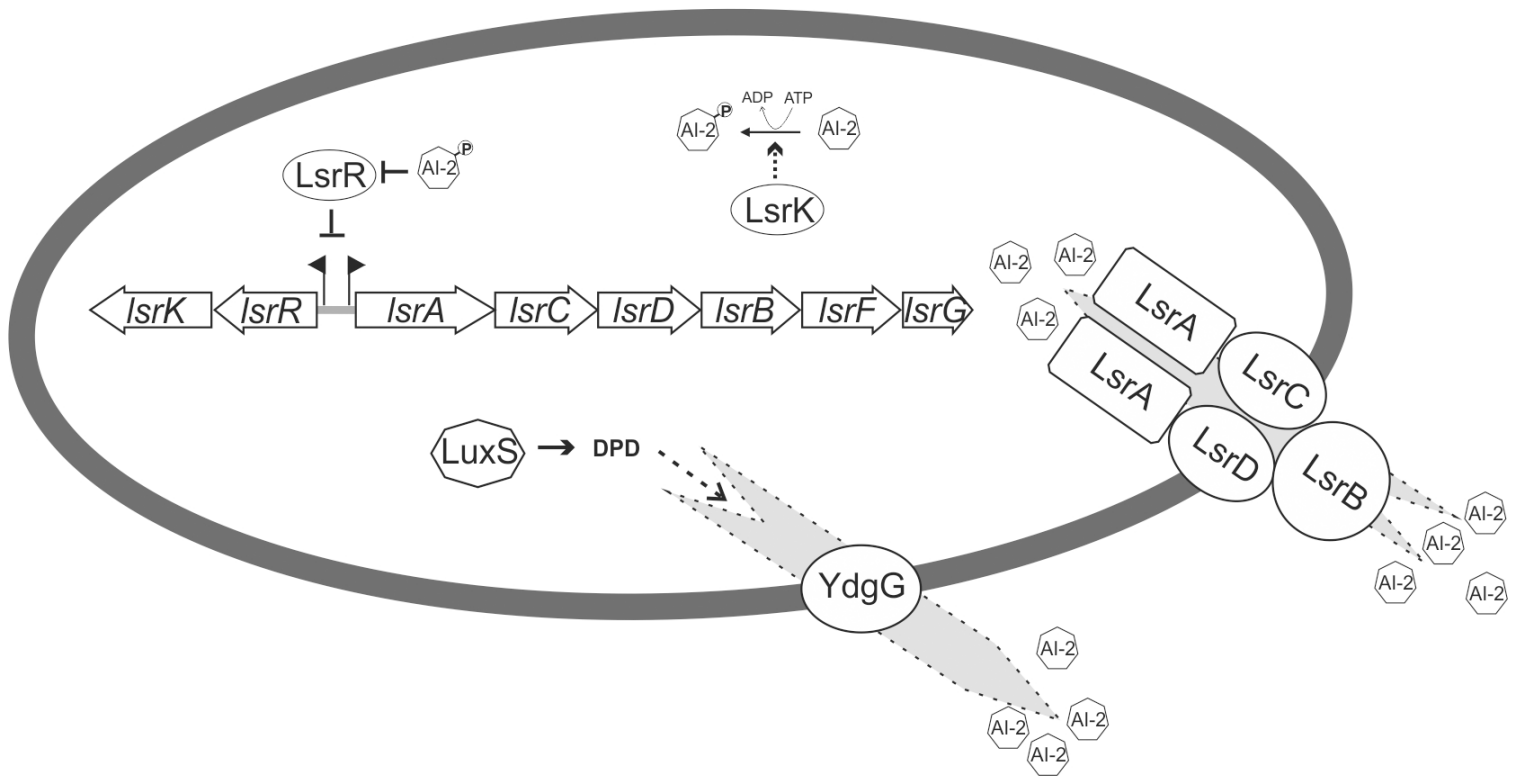
Regulatory circuit and Lsr-mediated transport and modification of AI-2 in *E. coli*. The AI-2 precursor DPD is produced by LuxS. AI-2 is then exported from the bacterial cell by YdgG. Extracellular AI-2 is imported by the ABC transporter LsrACDB. Intracellular AI-2 will be phosphorylated by the kinase LsrK and remains in the cytoplasm. The transcriptional repressor LsrR represses the expression of the *lsrACDBFG* and *lsrRK* transcriptional units by binding to their promoter region. Phosphorylated AI-2 can bind to the repressor LsrR and thereby relieve the transcriptional blockade. The subsequent expression of the LsrACDB transporter facilitates additional uptake of extracellular AI-2. Phosphorylated AI-2 is further processed by LsrG and LsrF.

In bacteria, the precursor of AI-2, (S)-4,5-dihydroxy-2,3-pentanedione (DPD) (Xavier and Bassler, 2005), is produced as a byproduct of L-homocysteine synthesis by the synthase LuxS, a widely distributed and conserved enzyme that is part of the activated methyl cycle (Schauder et al., 2001; Vendeville et al., 2005). DPD is a hydrophilic molecule that is actively transported out of the bacterial cell after production (Khera et al., 2022). Outside of the cell, DPD spontaneously circularizes into either boron-containing (S)-2-methyl2,3,3,4-tetrahydroxytetrahydrofuran-borate or boron-free (R)-2-methyl-2,3,3,4-tetrahydroxytetrahydrofuran (R-THMF), resulting in two different types of active AI-2 (Chen et al., 2002; Miller et al., 2004). Depending on the environmental status, DPD can spontaneously convert between the two active forms and thus be sensed by mixed bacterial communities since both AI-2 forms are perceived by different receptors (Zhang et al., 2020). R-THMF, from now on referred to as AI-2, is an intracellular signaling molecule (Xavier and Bassler, 2005). Different receptors for AI-2 are known, but the best-studied is LsrB. LsrB was first described in *Salmonella enterica* serovar Typhimurium (*S.* Typhimurium) (Taga et al., 2001) and is part of a specialized transporter encoded in the *lsr* (LuxS-regulated) locus (Taga et al., 2003). The *lsr* locus, or homologs, have been found in various bacterial species, including *E. coli* (Pereira et al., 2009), and comprises two transcription units. The first transcription unit, *lsrRK*, encodes the kinase LsrK that phosphorylates the incorporated AI-2 and the repressor LsrR that represses transcription of both *lsr* operons (Xue et al., 2009), as well as of several other genes in *E. coli* (Li et al., 2007) and *S.* Typhimurium (Choi et al., 2012). The binding of phosphorylated AI-2 to LsrR abolishes the transcriptional repression, resulting in AI-2-dependent gene expression (Xavier and Bassler, 2005). The second transcription unit, *lsrACDBFG*, encodes structural proteins of the AI-2 transporter, i.e., LsrA, LsrC, LsrD, and LsrB as the receptor, as well as the isomerase LsrG that degrades the phosphorylated AI-2 and the thiolase LsrF that catalyzes the terminal step in processing phosphorylated AI-2 (Marques et al., 2014) (**Figure 1**).

Interfering with AI-2-dependent QS, either by using QS inhibitors (Helmy et al., 2022) or gene mutations and deletions, led to altered behavior and lower virulence in ExPEC strains. For example, the deletion of *luxS* has led to a significant reduction of virulence in the APEC O78:K80:H9 strain χ7122 (Palaniyandi et al., 2013) and an attenuated virulence of the APEC strain DE17Δ*aroA* (Han et al., 2015). Also, the deletion of *lsrACD* in APEC94, which leads to an impaired AI-2 uptake and decreased bacterial motility, has led to lower bacterial loads and reduced virulence in a duckling infection model (Zuo et al., 2019). Additionally, it was shown that AI-2-dependent QS is associated with hydrogen peroxide (H_2_O_2_) resistance in the MPEC strain DCM5 (Wang et al., 2021) and antibiotic sensitivity in APEC strain APECX40 and the MPEC isolate DCM1 (Xue et al., 2016; Yu et al., 2020). Although AI-2-dependent QS has significant effects on APEC and MPEC, little is known about its effects on the virulence and fitness traits of UPEC, which are often genetically very similar to APEC and MPEC (Rodriguez-Siek et al., 2005; Tivendale et al., 2010). UPEC are the leading cause of urinary tract infections (UTI), one of the most prevalent bacterial infections worldwide, accounting for at least 75% of all complicated and uncomplicated cases (Flores-Mireles et al., 2015). Despite several host defense mechanisms, including the physicochemical composition of urine, the expression of antimicrobial peptides, or other innate immune response mechanisms (Abraham and Miao, 2015; Loubet et al., 2020), more than 50% of all women (Medina and Castillo-Pino, 2019) and about 20% of all men (Farrell et al., 2021) suffer at least from one symptomatic UTI episode during their lifetime. This indicates that UPEC strains are well adapted to the bladder environment and can combat the host’s defense strategies (Flores-Mireles et al., 2015; Mann et al., 2017).

The asymptomatic bacteriuria (ABU) *E. coli* isolate 83972 evolved from UPEC but has lost the ability to express many functional virulence factors (Zdziarski et al., 2008). This strain is well adapted to long-term bladder colonization without causing inflammation or other UTI-related symptoms (Hull et al., 1999). Additionally, *E. coli* 83972 has a better antioxidant defense than UPEC strains (Aubron et al., 2012) and can outcompete UPEC strains in human urine, amongst others, probably due to fast growth and nutritional adaptation (Ipe et al., 2016; George et al., 2024). Due to these characteristics, deliberate bladder colonization by *E. coli* 83972 has been suggested as a therapeutic approach to prevent recurring UTI by bacterial interference (Sundén et al., 2010; Köves et al., 2014; Wullt and Svanborg, 2016). Against the background of the increasing spread of multidrug-resistant bacteria, especially in the context of UTI, the use of antibiotics must be reduced (Cook and Wright, 2022). Bacterial interference by bladder colonization with ABU strain 83972 is an alternative way to prevent permanent colonization of the urinary bladder by symptomatic uropathogens (Wullt and Svanborg, 2016; Stork et al., 2018; Kenneally et al., 2022). Deeper insights into the importance of QS for the fitness and competitiveness of ABU strain 83972 may help to optimize the use of this bacterial strain for the deliberate therapeutic colonization of patients by uncovering underlying molecular mechanisms and thus improving the efficacy and safety of this form of treatment as an interesting alternative to antibiotic therapy. Interestingly, *E. coli* 83972 lacks the complete *lsr* locus but carries the *luxS* gene. We hypothesized that the absence of AI-2-dependent QS contributes to the strain’s fitness during bladder colonization. Therefore, we integrated the full-length *lsr* locus from the *E. coli* K-12 strain MG1655 into the chromosome of *E. coli* 83972 and analyzed the transcriptome of the *lsr* complemented strain to screen for differentially expressed genes that are affected by AI-2-dependent QS and may be relevant during bladder colonization. Additionally, we tested for relevant bacterial phenotypes that may increase this strain`s fitness in the urinary bladder.

## 2 Material and methods

Bacterial strains and culture conditions. The bacterial strains used in this study are listed in **Supplementary Table S5**. The asymptomatic bacteriuria *E. coli* isolate 83972 has been obtained as a gift from C. Svanborg (Lund, Sweden). Bacterial cultures were either cultivated in lysogeny broth (LB) (10 g/L tryptone, 5 g/L yeast extract, and 5 g/L NaCl) or pooled human urine (four male and six female voluntary individuals, sterile filtered and mixed in a 1:1 male/female ratio (v/v)). When appropriate, antibiotics were added in the following concentrations: kanamycin (25 µg/mL), chloramphenicol (12.5 µg/mL), ampicillin (100 µg/mL), or zeocin (50 µg/mL). Bacterial strains were grown overnight at 37°C on LB agar plates (containing 1.5% agar (w/v)) with the appropriate antibiotics when needed. For overnight cultures, single colonies were picked and incubated in 2 mL of LB at 37°C and with orbital shaking at 180 rpm. *E. coli* DH5α was used as a host for plasmid construction. Genome manipulation was done by recombineering (Datsenko and Wanner, 2000). When indicated, cultures were supplemented with H_2_O_2_ (stabilized, Merck Millipore, Darmstadt, Germany) or synthetic DPD purchased from Rita Ventura’s research group at ITQB-UNL (Oeiras, Portugal) (https://itqb.unl.pt/research/chemistry/bioorganic-chemistry). Synthetic DPD was synthesized as published before (Ascenso et al., 2011), and the concentration was 16.8 mM. Both chemicals were subdiluted to working concentrations using sterile ddH_2_O.

Cloning methods. DNA amplification for cloning and genetic manipulation was done using the Q5 High-Fidelity DNA Polymerase (New England Biolabs, Frankfurt/Main, Germany). Colony-PCR was done using the GoTaq Green Master Mix (Promega GmbH, Walldorf, Germany). After amplification, the correct size of amplicons was verified using agarose gel electrophoresis in 1x Tris-acetate-EDTA (TAE) buffer with 1-2% (w/v) agarose. The gels were run at 110–130 V for 30–60 min. Amplicons were purified using the NucleoSpin Gel and PCR Clean-up kit (Macherey-Nagel, Düren, Germany) or the Wizard SV Gel and PCR Clean-Up System kit (Promega, Walldorf, Germany). Plasmids were isolated using the NucleoSpin Plasmid Mini kit (Macherey-Nagel). Genomic DNA (gDNA) was isolated using the QIAamp DNA Mini kit (QIAGEN, Hilden, Germany). Purified PCR products were quantified using a spectrophotometer (NanoDrop 2000, Thermo Fisher Scientific, Schwerte, Germany). The restriction enzymes and T4 DNA ligase used for cloning were purchased from New England Biolabs (Frankfurt/Main, Germany).

Oligonucleotides and plasmids used. All oligonucleotides and plasmids used are listed in **Supplementary Tables S6**, **S7**.

Construction of pMK2. The plasmid pMK2 was constructed by integrating the *lsrA* promoter-*yfp* fusion reporter cassette coupled to *lsrR* (*lsrR*:P*lsrA*:*yfp* fusion) from the previously described *E. coli* K-12 strain MG1655 (*lsrA-G*::*yfp*-*cat*) (Keizers et al., 2022) into the low-copy number plasmid pWKS30 (Wang and Kushner, 1991). For that, the *lsrR*:P*lsrA*:*yfp* fusion was amplified from the gDNA of *E. coli* K-12 strain MG1655 (*lsrA-G*::*yfp*-*cat*) using the oligonucleotides pKD3_lsr_seq_4 and LZP50 and introduced into the *Sma*I-digested pWKS30 by using DNA T4 ligase. The correct insertion of the PCR product into pWKS30 was confirmed by plasmid preparation and subsequent Sanger sequencing.

Construction of pLS1 and pLS2. The low-copy-number plasmids pLS1 and pLS2 were constructed by integrating the P*dps*-*cfp* (*cfp* under the control of the stationary phase-dependent *dps* promoter) cassette or the P*dps*-*yfp* (*yfp* under the control of the stationary phase-dependent *dps* promoter) cassette, respectively, into pWKS30. Both cassettes were amplified using the oligonucleotides bla-TEM-r and MC_185 and pPS1 (P*dps*-*cfp*) or pPS2 (P*dps*-*yfp*) (Schiller et al., 2021) as templates. Amplicons and pWKS30 were cut with *Kpn*I and *Sma*I. All products were purified, and the digested P*dps*-*cfp* cassette was introduced into digested pWKS30, resulting in pLS1. The digested P*dps*-*yfp* cassette was introduced into digested pWKS30, resulting in pLS2. Ligation was done using DNA T4 ligase. Correct plasmids were confirmed by fluorescence microscopy.

Construction of *E. coli* strains 83972 Δ*luxS* and 83972 Δ*ybhC*. For the construction of *E. coli* 83972 Δ*luxS*, the *luxS* gene was replaced by a zeocin resistance cassette (*bleR*) that was amplified using the oligonucleotides Rec_BleoR_fwd and Rec_BleoR_rev and pEM7/Zeo as a template. Replacement was confirmed by colony PCR using the oligonucleotides CFT073_ΔluxS_CP_fwd and CFT073_ΔluxS_CP_rev and Sanger sequencing of the purified amplicon. For the construction of *E. coli* strain 83972 Δ*ybhC*, the *ybhC* gene was replaced by a chloramphenicol resistance cassette (*cat*) that was amplified using the oligonucleotides del_ybhC_fwd and del_ybhC_rev and pLP2 (Peng et al., 2022) as a template. Replacement was confirmed by colony PCR using the oligonucleotides wt_PybhC and LZP18 and Sanger sequencing of the purified amplicon.

Construction of *E. coli* strains 83972 *attB*::*lsr* and 83972 Δ*luxS attB:: lsr*. First, the plasmid pWKS30_LSR was generated. The chloramphenicol resistance cassette (*cat*) was introduced upstream of the *lsr* locus in the chromosome of *E. coli* strain MG1655 by recombineering (Datsenko and Wanner, 2000). The resistance cassette was amplified using the oligonucleotides CBL_fwd and CBL_rev and pMB54 (Berger et al., 2016) as a template. The correct chromosomal insertion was confirmed by colony PCR using the oligonucleotides LKRS_fwd and pMB54_lsrRK_CP1_rev. In the same way, the kanamycin resistance cassette (*aph*) was chromosomally inserted downstream of the *lsr* locus in the resulting *E. coli* strain MG1655 *cat*_*lsr*. The *aph* resistance cassette was amplified using the oligonucleotides RBL_fwd and RBL_rev and pKD4 as a template. The correct chromosomal insertion was confirmed by colony PCR using the oligonucleotides pKD3_lsr_seq_12 and LZP50. The full-length *lsr* locus, flanked by *cat* and *aph*, was amplified from gDNA of the resulting *E. coli* strain MG1655 *cat*_*lsr*_*aph* using the oligonucleotides LKRS_fwd and LZP50, and cloned into the *Sma*I-digested pWKS30 by using DNA T4 ligase. The relevant parts of the resulting plasmid pWKS30_LSR were afterward confirmed by Sanger sequencing. The complete *lsr* determinant was then inserted by recombineering (Datsenko and Wanner, 2000) next to the chromosomal attachment site of the bacteriophage λ (*attB*) in either *E. coli* 83972 or strain 83972 Δ*luxS*. For this, the *lsr* locus flanked by *cat* and *aph* (*cat*_*lsr*_*aph*) was amplified using the oligonucleotides pWKS30_attB_fwd and pWKS30_attB_rev and pWKS30_LSR as a template and introduced next to the λ *attB* site of the strains 83972 and 83972 Δ*luxS*, respectively. The correct chromosomal insertion was confirmed by colony PCR using the oligonucleotides CFT073_lsrRK_YFP_CP1_fwd and pKD3_lsr_seq_12 or CFT073_lsrRK_YFP_CP1_fwd and CFT073_lsrRK_YFP_CP2_rev and pKD3_lsr_seq_13. Afterward, gDNA was isolated, and the *cat*_*lsr*_*aph* region was amplified using the primer pair CFT073_lsrRK_YFP_CP1_fwd and CFT073_lsrRK_YFP_CP2_rev before the correctness of the DNA sequence was also verified by Sanger amplicon sequencing.

RNA isolation. Total RNA was isolated as previously described (Wallenstein et al., 2020) with the following differences: Overnight cultures of *E. coli* strains 83972 and 83972 *attB*::*lsr* were diluted to a final optical density OD600 = 0.02 in 200 mL LB. At indicated time points, 70 mL of culture (lag phase), 5 mL of culture (exponential phase), or 2 mL of culture (stationary phase) were added to the 0.2x volume of pre-chilled stop solution, and the bacterial pellets were directly lysed. Confirmation of complete gDNA removal was done by qPCR. RNA was extracted from three biological replicates for each strain.

**Figure 2:**
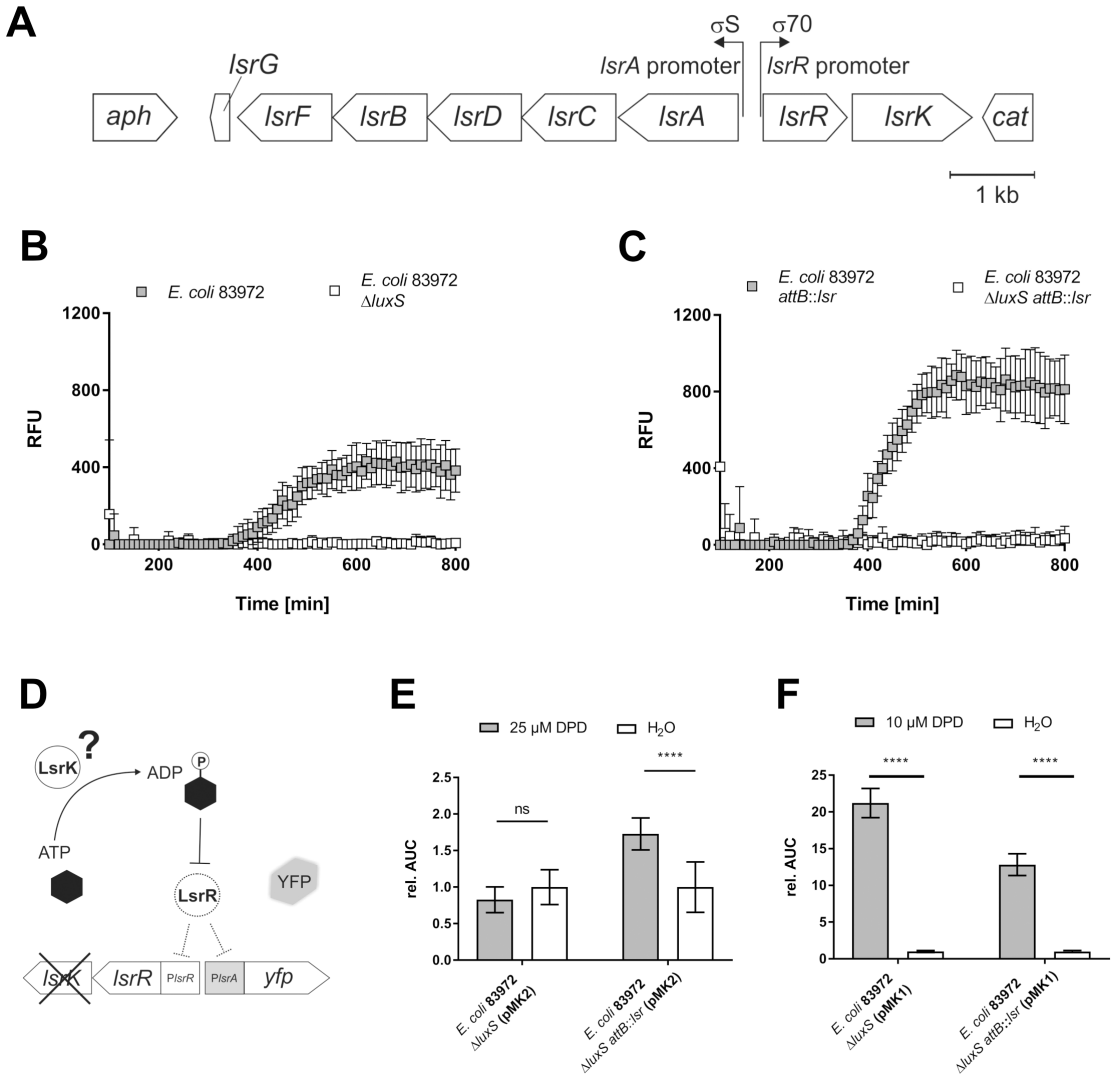
Complementation of *E*. *coli* 83972 with the *lsr* determinant and its impact on AI-2 synthesis, uptake and LsrK phosphorylation. The full-length *lsr* locus was introduced into *E*. *coli* 83972, and subsequent analyses revealed *luxS*-dependent AI-2 synthesis, the functionality of the complemented gene *lsrK* and the functionality of AI-2 uptake. **(A)** The *lsr* determinant flanked by two resistance cassettes (12,520 bp) was integrated into the chromosomal *attB* site of *E*. *coli* 83972. The *luxS*-dependent AI-2 synthesis was analyzed by fluorescence kinetics (100–800 min), depicted as RFU, during co-culture with the reporter strain *E*. *coli* Δ*luxS* (*attB*::P*lsrA-yfp*) (Keizers et al., 2022). Co-cultures consisting of the reporter strain and **(B)** either *E*. *coli* 83972 (grey squares) or *E*. *coli* 83972 Δ*luxS* (white squares) or **(C)** *E*. *coli* 83972 *attB*::*lsr* (grey squares) and *E*. *coli* 83972 Δ*luxS attB:: lsr* (white squares) in a 1:1 ratio in LB. Error bars represent the results of three biological replicates in duplicates each. **(D)** The functionality of the complemented gene *lsrK* was analyzed by incorporating the reporter plasmid pMK2 (comprises *lsrR* and the P*lsrA-yfp* reporter module) into *E*. *coli* strains 83972 Δ*luxS* and 83972 Δ*luxS attB*::*lsr*. YFP expression depends on AI-2 availability and the functional autoinducer-2 kinase LsrK. **(E)** Relative AUC calculation of P*lsrA* induction in *E*. *coli* strains 83972 Δ*luxS* (pMK2) and 83972 Δ*luxS attB*::*lsr* (pMK2) after the addition of 25 µM DPD compared to water during the late-exponential growth phase, as described before (Keizers et al., 2022). Statistical analysis was performed using the ordinary two-way ANOVA with Tukey’s multiple comparison test; values < 0.05 were considered statistically significant. Error bars represent the results of five biological replicates in duplicates each. **(F)** Relative AUC calculation of P*lsrA* induction in *E*. *coli* strains 83972 Δ*luxS* (pMK1) and 83972 Δ*luxS attB*::*lsr* (pMK1) after the addition of 10 µM DPD compared to water during the late-exponential growth phase, as described previously (Keizers et al., 2022). Statistical analysis was performed using Welch’s t-test; a value < 0.05 was considered statistically significant. Error bars represent the results of three biological replicates in duplicates each (ns, not significant, **** p<0.0001).

RNAseq and data analysis. Strand-specific cDNA libraries were prepared from the isolated RNA and sequenced (Illumina NextSeq 500, 1 x 75 bp single reads) by vertis Biotechnology AG (Freising, Germany). The obtained raw reads were analyzed as follows: Quality control using FastQC and MultiQC (Bioinformatics; Ewels et al., 2016), adaptor trimming using Cutadapt (Martin, 2011) with subsequent quality control, alignment of reads using burrows-wheeler alignment (Li and Durbin, 2009) to the genome of *E. coli* 83972 *attB*::*lsr* with a subsequent strandedness check using RSeQC (Wang et al., 2012) and quality control, count reading using featureCounts (Liao et al., 2014) and a subsequent differentially expressed gene analysis using DESeq2 (Love et al., 2014) in R. Conversion of formats was done using AGAT (Dainat, 2022). The subsequent data analysis was done in R. The Venn diagram (**Figure 2A**) was made using venn.diagram (Chen, 2022). The heatmaps (**Figures 2B–D**) were made using pheatmap (Kolde, 2019). Differentially expressed genes were clustered using dist() and hclust(), and biological replicates were clustered using pheatmap (cluster_cols = T) with the complete linkage method for hierarchial clustering. The scatterplots (**Supplementary Figure S2**) were computed using pairs.panels() from the package psych (Revelle, 2023).

Growth analysis and co-culture assays. For growth analysis, overnight cultures of the bacterial strains were diluted to a final optical density (OD600) of 0.01 in 1 mL of fresh medium with antibiotics when appropriate. 200 µL of freshly diluted cultures were added into one well of a 96-well plate (Thermo Fisher Scientific, Schwerte, Germany) in duplicates for each biological replicate. For the subsequent growth analysis, either an Infinite M NANO+ or an Infinite F200 microplate reader (both from TECAN, Männedorf, Switzerland) was used. The optical density was either measured at 595 nm (± 9 nm) (M NANO+) or 595 nm (± 10 nm) (F200). Signals were measured in ten-minute intervals. The resulting growth curves were analyzed using AMiGA (Analysis of Microbial Growth Assay (Midani et al., 2021)). For the lag phase length analysis, the data was not log-transformed. The co-culture assays were performed as previously described (Keizers et al., 2022). Briefly, overnight cultures of the strains to be analyzed were mixed in a 1:1 ratio with the *E. coli* K-12 reporter strain MG1655 Δ*luxS attB*::P*lsrA*-*yfp* to a final optical density (OD600) of 0.02. Growth analysis was performed as described above, and the fluorescence signal was either measured using an excitation wavelength of 514 nm (± 9 nm) and an emission wavelength of 550 nm (± 20 nm) (M NANO+) or using an excitation wavelength of 485 nm (± 20 nm) and an emission wavelength of 535 nm (± 25 nm) (F200).

Competition assays. For the competition assays, overnight cultures of *E. coli* strains 83972 (pLS2) and 83972 *attB*::*lsr* (pLS1) were mixed in a 1:1 ratio to a final optical density (OD600) of 0.02 in 25 mL of fresh medium supplemented with ampicillin. The cultures were grown at 37°C with orbital shaking at 180 rpm. After 3 h of growth, the cultures were subdiluted into 25 mL of fresh medium containing ampicillin with an appropriate dilution factor (1:200 for LB and 1:10 for pooled human urine). After subdilution, the cultures were grown for another 3 h. The subdilution procedure was repeated three times with a total assay time of 12 h. For the long-term competition, the cultures were grown at 37°C with orbital shaking at 180 rpm for 72 h. Directly after mixing and at the indicated time points, aliquots were taken from the cultures and microscopically analyzed using a Leica inverted microscope DMi8 with an attached camera at 400 x magnification. For each aliquot, ten microscopic pictures were taken with three channels each – Differential interference contrast (DIC), cyan fluorescent protein CFP (excitation 436 nm (± 20 nm); dichroic mirror 455 nm; emission 480 nm (± 40 nm) and fluorescein isothiocyanate (FITC) (excitation 480 nm (± 40 nm); dichroic mirror 505 nm; emission 527 nm (± 30 nm). Next, the number of bacteria per picture was quantified in the CFP channel and the FITC channel using an ImageJ script. The ratio of bacterial numbers in the FITC channel compared to the total bacterial numbers in both the FITC and CFP channels was calculated for each of the ten pictures, resulting in one mean ratio value with a standard deviation for each time point and biological replicate.

H_2_O_2_ resistance assay. For the H_2_O_2_ resistance assay, overnight cultures of the bacterial strains were diluted to a final optical density (OD600) of 0.01 in 1 mL of fresh medium. 190 µL of freshly diluted cultures were added into one well of a 96-well plate (Thermo Fisher Scientific) in duplicates for each biological replicate. After adding cultures, 10 µL of H_2_O_2_ diluted in ddH_2_O was added into each well, resulting in the indicated final H_2_O_2_ concentrations. The growth was analyzed directly after the addition using an Infinite M NANO+ plate reader (TECAN) or an Infinite F200 (TECAN). The optical density was either measured at 595 nm (± 9 nm) (M NANO+) or 595 nm (± 10 nm) (F200). Signals were measured in ten-minute intervals. The resulting growth curves were analyzed using AMiGA (Analysis of Microbial Growth Assay (Midani et al., 2021)). For the lag phase length analysis, the data was not log-transformed.

*Galleria mellonella* larvae feeding assay. *Galleria mellonella* larvae were purchased from Fauna Topics Zoobedarf Zucht und Handels GmbH (Marbach am Neckar, Germany) and reared on an artificial diet (22% maize meal, 22% wheat germ, 11% dry yeast, 17.5% beeswax, 11% honey, and 11% glycerin) as previously described (Mukherjee et al., 2020). To evaluate the fitness of the *E. coli* wild type strain 83972 and its *lsr*-complemented mutant in the larval digestive tract, overnight cultures (OD 600 = 1, grown in LB) of both strains were mixed in a 1:1 ratio and force-fed to sixth-instar larvae (weighing approximately 250–300 mg) (Lange et al., 2019). A 10-μL aliquot of this bacterial suspension was gently introduced into the larval mouth using 1-mL disposable syringes fitted with 0.4 x 20-mm needles mounted on a microapplicator. Control larvae received an equivalent volume (10 μL) of sterile LB. Following force-feeding, larvae were incubated at 37°C for 24 hours without food. All larvae survived the incubation period and were subsequently flash-frozen in liquid nitrogen, ground into a fine powder, and homogenized in LB. The homogenates were then plated onto LB agar, and colony-forming units (CFUs) were counted following a 24-hour incubation at 37°C. The survival of the wild type and *lsr*-complemented mutant strains was analyzed by comparing their initial inoculation ratio to the ratio recovered from larvae after 24 hours of incubation.

Detection of the *lsr* determinant in *E. coli* genomes. To screen for the presence and conservation of the *lsr* determinant in *E. coli*, all published *E. coli* genomes that were available at the time point of the analysis were downloaded from NCBI Assembly (Kitts et al., 2016) (time point of download: 15.03.2023) in FASTA format (Status: latest RefSeq). In total, 32,594 genomes were downloaded. Next, the phylogroup was determined using ClermonTyping (Beghain et al., 2018) for each genome. The analysis was continued with 32,404 genomes belonging to the phylogroups A, B1, B2, C, D, E, F, and G. Next, each genome was analyzed using the basic local alignment search tool (BLAST+ (Camacho et al., 2009)) to screen for the nucleotide sequence (blastn) of the full-length *lsr* locus (*lsrRK*-*lsrACDBFG*), the *luxS* gene and the *tam* gene (reference sequences from the *E. coli* K-12 strain MG1655). Output possibilities were “complete” (blastn hit length equal query length), “incomplete” (blastn hit length inequal query length), “no match” (no blastn hit) or “multiple matches” (multiple blastn hits). The output “< 100 bp” is a summary of a blastn hit length of below 100 bp and the “no match” hits. Please note that our analysis only focuses on gene length, whereas the functionality of the encoded gene product was not part of the analysis.

Statistical analysis. Statistical analysis was performed using GraphPad Prism v8.0.2 (San Diego, USA).

## 3 Results

### 3.1 Complementation of *E. coli* 83972 with the *lsr* locus: impact on AI-2 uptake, synthesis and secretion

We complemented *E. coli* strain 83972 with the *lsr* locus by integrating the full-length *lsr* determinant (*lsrACDBFG* and *lsrRK* operons), flanked by two resistance markers, into the chromosomal attachment site (*attB*) of the bacteriophage λ (**Figure 2A**). The resulting *E. coli* strain 83972 *attB*::*lsr*, the wild type strain 83972, as well as their isogenic *luxS* deletion mutants (83972 Δ*luxS* and 83972 Δ*luxS attB*::*lsr*), were used for further analysis. The ability of strains 83972 and 83972 *attB*::*lsr* to secrete AI-2 was evaluated in co-cultures with the reporter strain *E. coli* Δ*luxS* (*attB*::P*lsrA*-*yfp*), as described previously (Keizers et al., 2022). We detected AI-2-dependent YFP-expression in the co-cultures with *E. coli* 83972 and *E. coli* 83972 *attB*::*lsr*, whereas we observed no YFP-expression in co-cultures with the isogenic *luxS* deletion strains. Both strains 83972 and 83972 *attB*::*lsr* synthesized AI-2in a *luxS*-dependent manner and exported it, which can be sensed by the P*lsrA* reporter strain in the co-culture. In the presence of the *lsr* determinant, *E. coli* 83972 can accumulate additional AI-2, leading to a stronger *lsrA* promoter induction in the reporter strain (**Figures 2B, C**).

To test if the *lsr*-complemented strain was capable of taking up and phosphorylating AI-2, we introduced pMK2, a derivative of the previously described reporter plasmid pMK1 that lacks *lsrK* (Keizers et al., 2022), into the strains 83972 Δ*luxS* and 83972 Δ*luxS attB*::*lsr* (**Figure 2D**). The functionality of LsrK was analyzed by supplementing the growth medium with 25 µM synthetic DPD in the late logarithmic growth phase and comparing the AI-2-dependent YFP expression to a water control. There was no significant difference in YFP expression between *E. coli* 83972 Δ*luxS* (pMK2) supplemented with 25 µM DPD and the water control. The expression of the AI-2 kinase LsrK in *E. coli* 83972 Δ*luxS attB*::*lsr* (pMK1) led to a significantly higher YFP expression when the strain was supplemented with 25 µM DPD as compared to the water control (p < 0.001) (**Figure 2E**). To determine if the observed differences were due to an overall defect in AI-2 uptake in the *lsr*-negative 83972 wild type strain, we introduced the reporter plasmid pMK1 into *E. coli* 83972 Δ*luxS* and *E. coli* 83972 Δ*luxS attB*::*lsr*. Again, AI-2 uptake was analyzed by supplementing the medium with 10 µM synthetic DPD in the late logarithmic growth phase and comparing the AI-2-dependent YFP expression to a water control. In both strains harboring pMK1, we detected a significantly higher YFP expression after the addition of 10 µM DPD relative to the corresponding water controls (p < 0.001). This confirmed that external AI-2 can be taken up by *E. coli* 83972 in an LsrACBD-independent way (**Figure 2F**).

### 3.2 Influence of the *lsr* determinant on global gene expression in *E. coli* 83972

**Figure 3:**
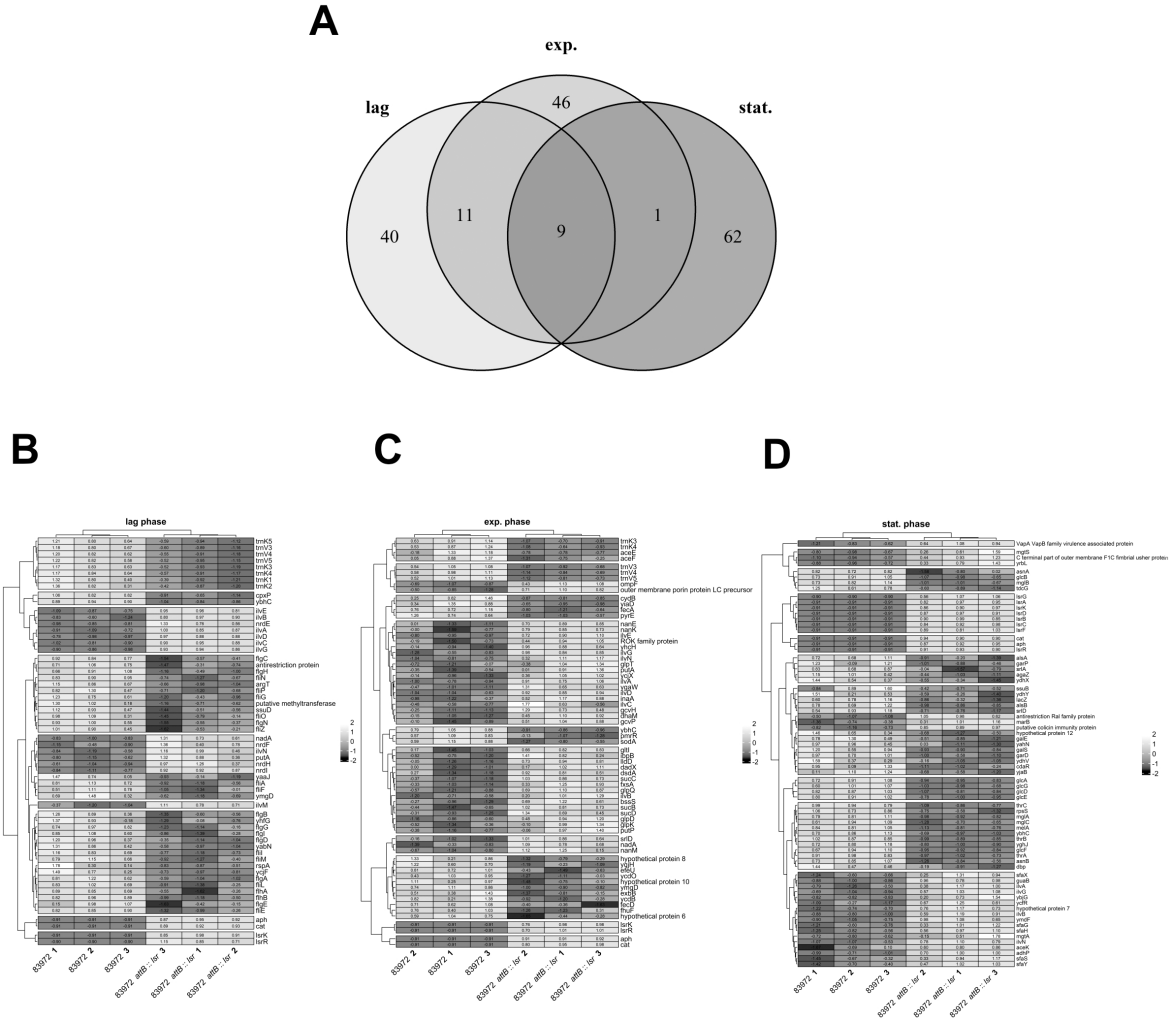
Differentially expressed genes in *E*. *coli* 83972 *attB*::*lsr* compared to its *lsr*-negative parental strain 83972. **(A)** The Venn diagram indicates the number of differentially expressed genes (DEG) in the lag phase (lag), mid-exponential phase (exp.) and stationary phase (stat.). The heatmaps depict the groups of genes which are differentially expressed in an *lsr*-dependent manner in **(B)** lag phase, **(C)** exp. phase and **(D)** stat. phase.

To analyze the effect of AI-2-dependent quorum sensing on the transcriptome, we performed an RNA-seq analysis of *E. coli* strains 83972 and 83972 *attB*::*lsr*. Both strains were cultivated in LB, and samples were taken in the lag phase, during mid-exponential growth (exp. phase), and during the transition to the stationary phase (stat. phase) (**Supplementary Figure S1**). We isolated total RNA from three biological replicates of each strain and time point for RNAseq analysis. For each library, we obtained ~ 11.5 x 10^6^ reads (± 2 x 10^6^ reads), from which, on average, ~ 89% (± 3-5%) were mapped to the reference genome (*E. coli* 83972 *attB*::*lsr*). Data analysis was done using DESeq2 in R (Love et al., 2014), and the resulting expression data was used for further analysis (**Supplementary Figure S2**). The transcriptome analysis revealed 492 significantly differentially expressed genes (DEG; adjusted p-value < 0.05) in *E. coli* 83972 *attB*::*lsr* compared to the wild type strain 83972 in the three growth phases. A complete list of all DEGs is provided in the **Supplementary Table S1**. Of the 492 DEGs, 199 genes had a log2-fold change (L2FC) in expression of at least 1 (DEG (± 1 L2FC)). Among all DEG (± 1 L2FC), nine genes were differentially expressed during all three growth phases (**Figure 3A**). We found 60 DEG (± 1 L2FC) in the lag phase, 67 DEG (± 1 L2FC) during mid-exponential growth, and 72 DEG (± 1 L2FC) during the transition to the stationary phase (**Figures 3B–D**). Besides *lsrR*, *lsrK* and the two resistance cassettes *aph* and *cat* that were used to complement *E. coli* 83972 *attB*::*lsr*, genes related to the isoleucine/valine biosynthesis pathway, namely *ilvNBAG*, were upregulated, and the gene *ybhC*, which encodes an uncharacterized protein that was suggested to be involved in H_2_O_2_ resistance, was downregulated in all three growth phases. The DEG (± 1 L2FC) can be functionally clustered into four main groups, i.e., metabolism, motility/biofilm formation/adhesion, growth and stress response, and some remaining genes (**Tables 1**–**5**).

**Table 1:** Differentially expressed genes in *E. coli* 83972 *attB*::*lsr* relative to *E. coli* 83972 associated with metabolism.

Gene name	log2-fold change	Pathway/function ^82–84^
lag phase		
*ybhC*	-3.442713222	pentose and glucuronate interconversions
*ssuD*	-1.736408299	sulfur metabolism
*argT*	-1.686346996	lysine/arginine/ornithine ABC transporter
*yaaJ*	-1.090400775	alanine or glycine:cation symporter
*rspA*	-1.020974991	pentose and glucuronate interconversions
*putA*	1.245756938	alanine, aspartate and glutamate metabolism; arginine and proline metabolism
*ilvN*	1.272441162	butanoate metabolism; C5-Branched dibasic acid metabolism/valine, leucine and isoleucine biosynthesis; pantothenate and CoA biosynthesis
*nadA*	1.292375467	nicotinate and nicotinamide metabolism
*ilvB*	1.403448526	butanoate metabolism; C5-Branched dibasic acid metabolism; valine, leucine and isoleucine biosynthesis; pantothenate and CoA biosynthesis
*ilvA*	2.26260477	glycine, serine and threonine metabolism; valine, leucine and isoleucine biosynthesis
*ilvM*	2.345314152	butanoate metabolism; C5-Branched dibasic acid metabolism; valine, leucine and isoleucine biosynthesis; pantothenate and CoA biosynthesis
*ilvD*	2.459224415	valine, leucine and isoleucine biosynthesis; pantothenate and CoA biosynthesis
*ilvE*	2.660495823	cysteine and methionine metabolism; valine, leucine and isoleucine degradation; valine, leucine and isoleucine biosynthesis; pantothenate and CoA biosynthesis
*ilvG*	3.063866229	butanoate metabolism; C5-Branched dibasic acid metabolism; valine, leucine and isoleucine biosynthesis; pantothenate and CoA biosynthesis
*ilvC*	4.371339203	valine, leucine and isoleucine biosynthesis; pantothenate and CoA biosynthesis
exp. phase		
*ybhC*	-3.005651583	pentose and glucuronate interconversions
*fhuF*	-2.013029339	ferric iron reductase
*fecD*	-1.925348313	iron-dicitrate ABC transporter subunit
*aceE*	-1.523183226	glycolysis; gluconeogenesis; citrate cycle (TCA cycle); pyruvate metabolism; lipoic acid metabolism
*ycdB*	-1.472910514	iron uptake system component
*aceF*	-1.398523034	glycolysis; gluconeogenesis; citrate cycle (TCA cycle); pyruvate metabolism; lipoic acid metabolism
*ycdO*	-1.354592654	iron uptake system component
*yqjH*	-1.302729542	ferric-chelate reductase
*efeU*	-1.289042204	ferrous iron permease
*fecA*	-1.188669015	ferric citrate outer membrane transporter
*exbB*	-1.172171856	enterochelin uptake protein
*sucB*	1.003798232	citrate cycle (TCA cycle); lysine degradation; tryptophan metabolism; lipoic acid metabolism
*dhaM*	1.010654719	glycerolipid metabolism
*gcvP*	1.084269904	glyoxylate and dicarboxylate metabolism; glycine, serine and threonine metabolism; lipoic acid metabolism
*ilvC*	1.225445888	valine, leucine and isoleucine biosynthesis; pantothenate and CoA biosynthesis
*glpK*	1.237027056	glycerolipid metabolism
ROK family protein	1.2491515	amino sugar and nucleotide sugar metabolism
*dsdA*	1.265931194	glycine, serine and threonine metabolism; D-Amino acid metabolism
*sucC*	1.271455252	citrate cycle (TCA cycle); propanoate metabolism; C5-Branched dibasic acid metabolism
*putP*	1.326926235	sodium/proline symporter
*gltI*	1.330564322	glutamate/aspartate transport system
*nadA*	1.36410938	nicotinate and nicotinamide metabolism
*glpT*	1.367466661	glycerol-3-phosphate transporter
*sucD*	1.382561251	citrate cycle (TCA cycle); propanoate metabolism; C5-Branched dibasic acid metabolism
*ygaW*	1.400831967	L-Alanine exporter
*glpQ*	1.436168876	glycerophospholipid metabolism
*glpD*	1.473785806	glycerophospholipid metabolism
*nanM*	1.493614699	N-acetylneuraminate epimerase
*gcvH*	1.5078413	glyoxylate and dicarboxylate metabolism; glycine, serine and threonine metabolism; lipoic acid metabolism
*ilvD*	1.775787503	valine, leucine and isoleucine biosynthesis; pantothenate and CoA biosynthesis
*ilvA*	1.784324762	glycine, serine and threonine metabolism; valine, leucine and isoleucine biosynthesis
*dadX*	1.812119174	D-Amino acid metabolism
*srlD*	1.819297687	fructose and mannose metabolism
*putA*	1.857930281	alanine, aspartate and glutamate metabolism; arginine and proline metabolism
*nanK*	2.039366083	amino sugar and nucleotide sugar metabolism
*lldD*	2.043637543	pyruvate metabolism
*nanE*	2.076864669	amino sugar and nucleotide sugar metabolism
*ilvE*	2.167676205	cysteine and methionine metabolism; valine, leucine and isoleucine degradation; valine, leucine and isoleucine biosynthesis; pantothenate and CoA biosynthesis
*ilvB*	2.647462771	butanoate metabolism; C5-Branched dibasic acid metabolism; valine, leucine and isoleucine biosynthesis; pantothenate and CoA biosynthesis
*yhcH*	2.806747795	N-acetylneuraminate anomerase
*ilvG*	3.332537625	butanoate metabolism; C5-Branched dibasic acid metabolism; valine, leucine and isoleucine biosynthesis; pantothenate and CoA biosynthesis
*ilvN*	3.406210862	butanoate metabolism; C5-Branched dibasic acid metabolism; valine, leucine and isoleucine biosynthesis; pantothenate and CoA biosynthesis
stat. phase		
*glcE*	-5.809515048	glyoxylate and dicarboxylate metabolism
*glcD*	-5.508308467	glyoxylate and dicarboxylate metabolism
*glcA*	-3.84055795	glycolate permease
*glcG*	-3.437418583	glycolate utilization
*glcB*	-3.040428269	pyruvate metabolism; glyoxylate and dicarboxylate metabolism
*alsA*	-2.970438825	D-allose transport ATP-binding protein
*glcF*	-2.686837435	glyoxylate and dicarboxylate metabolism
*garP*	-2.178038147	predicted (D)-galactarate transporter
*thrC*	-2.009914989	glycine, serine and threonine metabolism; vitamin B6 metabolism
*thrB*	-1.883274845	glycine, serine and threonine metabolism
*thrA*	-1.856342407	glycine, serine and threonine metabolism; cysteine and methionine metabolism; lysine biosynthesis; monobactam biosynthesis
*tdcG*	-1.747142565	glycine, serine and threonine metabolism; cysteine and methionine metabolism
*agaZ*	-1.742650224	galactose metabolism
*alsB*	-1.728052629	D-allose transporter subunit
*galS*	-1.637606696	transcriptional repressor and galactose ultrainduction factor
*garD*	-1.620616567	ascorbate and aldarate metabolism
*srlA*	-1.61111755	fructose and mannose metabolism
*ybhC*	-1.567162144	pentose and glucuronate interconversions
*mglB*	-1.542837699	D-galactose-binding periplasmic protein precursor
*mglA*	-1.531637382	ABC-type D-galactose transporter
*asnA*	-1.500124244	alanine, aspartate and glutamate metabolism; cyanoamino acid metabolism
*ssuB*	-1.413738896	sulfur metabolism
*srlD*	-1.38914946	fructose and mannose metabolism
*galE*	-1.350506556	galactose metabolism; amino sugar and nucleotide sugar metabolism; O-Antigen nucleotide sugar biosynthesis
*mglC*	-1.329707605	galactoside transport system permease protein
*melA*	-1.18645874	galactose metabolism; glycerolipid metabolism; sphingolipid metabolism
*asnB*	-1.065620685	alanine, aspartate and glutamate metabolism
*cdaR*	-1.061008612	carbohydrate diacid regulator
*lacZ*	-1.015924833	galactose metabolism; sphingolipid metabolism; other glycan degradation
*yahN*	-1.003789478	amino acid exporter
*adhP*	1.015337508	glycolysis; gluconeogenesis; pyruvate metabolism; fatty acid degradation; tyrosine metabolism; chloroalkane and chloroalkene degradation; naphthalene degradation
*guaB*	1.019960645	purine metabolism
*ilvA*	1.074184986	glycine, serine and threonine metabolism; valine, leucine and isoleucine biosynthesis
*aceK*	1.192060758	isocitrate dehydrogenase kinase/phosphatase
*ilvB*	1.778668589	butanoate metabolism; C5-Branched dibasic acid metabolism; valine, leucine and isoleucine biosynthesis; pantothenate and CoA biosynthesis
*mgtS*	1.992512123	Mg2^+^-import associated protein
*ilvG*	2.430932647	butanoate metabolism; C5-Branched dibasic acid metabolism; valine, leucine and isoleucine biosynthesis; pantothenate and CoA biosynthesis
*ilvN*	2.670495316	butanoate metabolism; C5-Branched dibasic acid metabolism; valine, leucine and isoleucine biosynthesis; pantothenate and CoA biosynthesis
*mgtA*	2.724809679	Mg2^+^-importing ATPase

**Table 2:** Differentially expressed genes in *E. coli* 83972 *attB*::*lsr* relative to *E. coli* 83972 associated with motility, biofilm formation and adhesion.

Gene name	log2-fold change	Pathway/function ^82–84^
lag phase		
*fliN*	-2.453087846	bacterial chemotaxis; flagellar assembly
*flgC*	-2.370643137	flagellar assembly
*fliG*	-2.154193018	bacterial chemotaxis; flagellar assembly
*fliA*	-1.967650727	biofilm formation; flagellar assembly
*fliP*	-1.934071506	flagellar assembly
*flgG*	-1.89377638	flagellar assembly
*fliE*	-1.817157817	flagellar assembly
*fliO*	-1.802383017	flagellar assembly
*flgN*	-1.76655253	flagellar assembly
*flgH*	-1.748868638	flagellar assembly
*flgB*	-1.704583646	flagellar assembly
*fliM*	-1.650792441	bacterial chemotaxis; flagellar assembly
*fliL*	-1.611497848	flagellar assembly
*flhB*	-1.518230416	flagellar assembly
*flgA*	-1.464314687	flagellar assembly
*flgD*	-1.426698341	flagellar assembly
*flgI*	-1.32985639	flagellar assembly
*fliZ*	-1.324136977	biofilm formation; flagellar assembly
*fliI*	-1.274724031	flagellar assembly
*flhA*	-1.24886028	flagellar assembly
*flgE*	-1.199949551	flagellar assembly
*fliF*	-1.178707702	flagellar assembly
exp. phase		
*bssS*	1.02819806	biofilm regulator; TA system
stat. phase		
*sfaX*	1.107129346	transcriptional regulator; S-fimbrial assembly
C terminal part of outer membrane F1C fimbrial usher protein SfaF	1.280095448	S-fimbrial assembly
*sfaS*	1.317340212	secretion system; S-fimbrial assembly
*sfaG*	1.364663339	adhesion; S-fimbrial assembly
*sfaY*	1.429358699	transcriptional regulator; S-fimbrial assembly
*sfaH*	1.475091904	secretion system; S-fimbrial assembly

**Table 3:** Differentially expressed genes in *E. coli* 83972 *attB*::*lsr* relative to *E. coli* 83972 associated with growth.

Gene name	log2-fold change	Pathway/function ^82–84^
lag phase		
*trnK3*	-1.936591094	aminoacyl-tRNA biosynthesis
*trnK4*	-1.904947039	aminoacyl-tRNA biosynthesis
*trnV3*	-1.772558046	aminoacyl-tRNA biosynthesis
*trnV5*	-1.713113393	aminoacyl-tRNA biosynthesis
*trnV4*	-1.708725917	aminoacyl-tRNA biosynthesis
*trnK1*	-1.324258994	aminoacyl-tRNA biosynthesis
*trnK2*	-1.217243916	aminoacyl-tRNA biosynthesis
*trnK5*	-1.150592884	aminoacyl-tRNA biosynthesis
*nrdF*	1.0340548	deoxyribonucleotide biosynthesis
*nrdH*	1.139857909	deoxyribonucleotide biosynthesis
*nrdE*	1.269219097	deoxyribonucleotide biosynthesis
*nrdI*	1.30323686	deoxyribonucleotide biosynthesis
exp. phase		
*trnK3*	-1.676797599	aminoacyl-tRNA biosynthesis
*trnK4*	-1.60586492	aminoacyl-tRNA biosynthesis
*trnV4*	-1.238175595	aminoacyl-tRNA biosynthesis
*trnV5*	-1.23717919	aminoacyl-tRNA biosynthesis
*trnV3*	-1.237008793	aminoacyl-tRNA biosynthesis
*pyrE*	-1.194313829	pyrimidine *de novo* biosynthesis
*cydB*	-1.006975401	cytochrome d ubiquinol oxidase subunit
stat. phase		
*rpsS*	-1.193234994	component of 30S ribosomal subunit
*dbpA*	-1.087482054	ribosome biogenesis
*ybjG*	1.166583523	peptidoglycan biosynthesis; teichoic acid biosynthesis

**Table 4:** Differentially expressed genes in *E. coli* 83972 *attB*::*lsr* relative to *E. coli* 83972 associated with stress response.

gene name	log2-fold change	pathway/function ^82–84^
lag phase		
*cpxP*	-1.012637356	chaperones and folding catalysts; resistance to extracytoplasmic stress
exp. phase		
*sodA*	-1.132854292	superoxide dismutase
*ycjX*	1.056824975	induced under nitrogen starvation
*inaA*	1.254017352	pH-inducible protein involved in stress response
*ibpB*	2.077026895	heat shock protein
stat. phase		
*ydhY*	-1.674500305	predicted ferredoxin-like protein
*ydhX*	-1.481012743	predicted ferredoxin-like protein
*ydhV*	-1.131341197	aldehyde ferredoxin oxidoreductase
*marB*	1.133272934	multiple antibiotic resistance protein
*ymdF*	1.250735552	stress-induced protein
*ycfR*	1.262325635	stress-response protein

**Table 5:** Remaining differentially expressed genes in *E. coli* 83972 *attB*::*lsr* relative to *E. coli* 83972.

Gene name	log2-fold change	Pathway/function ^82–84^
lag phase		
putative methyltransferase	-2.044036679	–
antirestriction protein	-1.674016348	–
*yabN*	-1.441756062	transcription factor
*yhfG*	-1.409124762	TA System
*ycjF*	-1.10548474	predicted membrane protein associated with virulence in a murine model
*ymgD*	-1.024673712	possibly TA system
*lsrR*	6.259241476	transcriptional repressor; *lsr* locus introduction
*lsrK*	6.922763675	AI-2 kinase; *lsr* locus introduction
*aph*	11.20318319	kanamycin resistance gene; *lsr* locus introduction
*cat*	11.59026441	chloramphenicol resistance gene; *lsr* locus introduction
exp. phase		
*ymgD*	-2.028703146	possibly TA system
hypothetical protein 10	-1.823290623	–
hypothetical protein 6	-1.662041435	–
hypothetical protein 8	-1.33921833	–
*yiaD*	-1.267749645	putative outer membrane protein
*pmrR*	-1.155320974	small regulatory membrane protein
outer membrane porin protein LC precursor	1.100938115	transporter; pores iron channel
*fxsA*	1.275237522	cytoplasmic membrane protein
*ompF*	1.328798394	outer membrane protein
*lsrR*	8.021393949	transcriptional repressor; *lsr* locus introduction
*lsrK*	8.277667483	AI-2 kinase; *lsr* locus introduction
*aph*	11.13917456	kanamycin resistance gene; *lsr* locus introduction
*cat*	13.30014141	chloramphenicol resistance gene; *lsr* locus introduction
stat. phase		
*yghJ*	-2.038713336	putative lipoprotein
*yjaB*	-1.080319092	peptidyl-lysine *N*-acetyltransferase
hypothetical protein 12	-1.031550868	–
*yrbL*	1.010712982	–
antirestriction Ral family protein	1.033440867	–
putative colicin immunity protein	1.061726625	–
hypothetical protein 7	1.370170227	–
VapA/VapB family virulence associated protein	1.892144739	–
*lsrG*	6.851703901	isomerase; *lsr* locus introduction
*lsrC*	8.029960122	part of AI-2 transporter; *lsr* locus introduction
*lsrB*	8.088569874	AI-2 receptor; *lsr* locus introduction
*lsrF*	8.122185059	thiolase; *lsr* locus introduction
*lsrD*	8.795794664	part of AI-2 transporter; *lsr* locus introduction
*lsrA*	9.402359596	part of AI-2 transporter; *lsr* locus introduction
*lsrK*	9.814535263	AI-2 kinase; *lsr* locus introduction
*lsrR*	10.53084923	transcriptional repressor; *lsr* locus introduction
*aph*	11.52317813	kanamycin resistance gene; *lsr* locus introduction
*cat*	13.6273497	chloramphenicol resistance gene; *lsr* locus introduction

In the lag phase, mainly biosynthesis genes were upregulated. Together with the genes of the valine, leucine, and isoleucine biosynthesis pathway (*ilvNBAMDEGC*), also genes of the *nrd* operon, encoding for a ribonucleotide reductase (*nrdHIEF*) were significantly upregulated. On the contrary, several metabolic genes were downregulated. We found that genes involved in arginine uptake and metabolism (*argT*), sulfur utilization (*ssuD*), or coding for an alanine/glycine:cation symporter (*yaaJ*), as well as a mannonate dehydratase (*rspA*) were downregulated. Also, the expression of genes associated with the aminoacyl-tRNA biosynthesis of lysine (*trnK*1-5) and valine (*trnV*3-5) or with resistance against extracytoplasmic stress (*cpxP*) was downregulated. Amongst all genes that were downregulated in strain 83972 in the presence of the *lsr* determinant in the lag phase, more than 50% code for components of the flagellar assembly apparatus. In addition to the flagellar sigma factor σ28-encoding gene *fliA*, the transcriptional regulator gene *fliZ*, and the chaperone gene *flgN*, nearly all flagellar genes belonging to class 2 were downregulated (*fliE*, *fliFG*, *fliI*, *fliLMNOP*, *flhBA*, *flgBCDE*, *flgGHI*, and *flgA*).

During mid-exponential growth, we predominantly found genes associated with metabolic pathways upregulated in *E. coli* 83972 *attB*::*lsr* relative to *E. coli* strain 83972. The encoded gene products are involved in the metabolism of D-serine (*dsdA*), glutamate (*gltI*), glycine (*gcvPH*), L-lactate (*lldD*), sorbitol (*srlD*), L-alanine (*dadX*, *ygaW*), glycerol (*glpDTQK*), proline (*putAP*), N-acetylneuraminate (*nanMKE*-*yhcH*), dihydroxyacetone (*dhaM*) as well as the TCA cycle (*sucBCD*). Also, three genes encoding outer membrane proteins (*ompF* and the outer membrane porin protein LC precursor) were upregulated. The genes coding for the valine, leucine, and isoleucine biosynthesis pathway (*ilvNBADEGC*) were upregulated. Interestingly, we also found upregulated genes involved in stress response, including the nitrogen starvation gene *ycjX*, the heat-shock protein gene *ibpB*, and the pH-stress response protein gene *inaA*. On the contrary, the gene cluster *aceEF*, encoding a pyruvate dehydrogenase, was downregulated during mid-exponential growth. Of all downregulated genes during mid-exponential growth, 36% are associated with iron sensing and utilization, including the genes coding for the ferric siderophore reductase (*fhuF*), the ferric chelate reductase (*yqjH*), the ferric citrate transporter genes (*fecA*) and (*fecD*), the ferrous iron permease and transport genes *efeU*, *ycdB*, and *ycdO*, the energy transducing Ton complex subunit gene *exbB* and *pmrR*, which is associated with the iron and acidic pH sensing BasSR complex. Additionally, apart from *ybhC*, the superoxide dismutase SodA-encoding gene was downregulated. The latter two proteins have been described to protect against oxidative stress.

As expected, we observed that the genes of the *lsr* locus were highly expressed during the transition to the stationary phase. Also, the expression of valine, leucine, and isoleucine biosynthesis pathway genes (*ilvGABN*) and genes contributing to magnesium sensing and utilization were upregulated. These included the import-associated genes *mgtA* and *mgtS* and the Mg^2+^-induced kinase gene *yrbL*. Interestingly, several genes associated with general stress response were upregulated. These included *marB*, encoding the protein MarB that reduces the transcription rate of *marA*, which codes for a pleiotropic regulator also involved in general stress response. The expression of *ycfR*, which is involved in stress response and outer membrane permeability was also upregulated. We also detected that the transcript levels of several genes (*focDFGHYX*) of the F1C fimbrial operon were significantly increased in *E. coli* 83972 *attB*::*lsr* in comparison to *E. coli* strain 83972. The main proportion of downregulated genes, was related to metabolism. The *yghJ* (*sslE*) gene, for example, codes for a metalloprotease contributing to mucin degradation in the intestinal tract or the bladder (Nesta et al., 2014). The most strongly downregulated genes were linked to glycolate utilization (*glcDEFGBA*). Additionally, genes that are involved in D-allose transport (*alsAB*), galactarate metabolism (*garPD* and *cdaR*), galactose metabolism (*mglABC*, *lacZ*, *melA*, *agaZ* and *galES*), sorbitol utilization (*srlAD*), D-serine metabolism (*tdcG*) and sulfur utilization (*ssuB*) were significantly downregulated, too. Furthermore, genes associated with L-threonine (*thrABC*) and asparagine (*asnAB*) synthesis, as well as three genes that code for components of a putative oxidoreductase (*ydhYVX*) were also downregulated.

### 3.3 Reintroduction of the *lsr* locus reduces competitiveness *in vitro*

**Figure 4:**
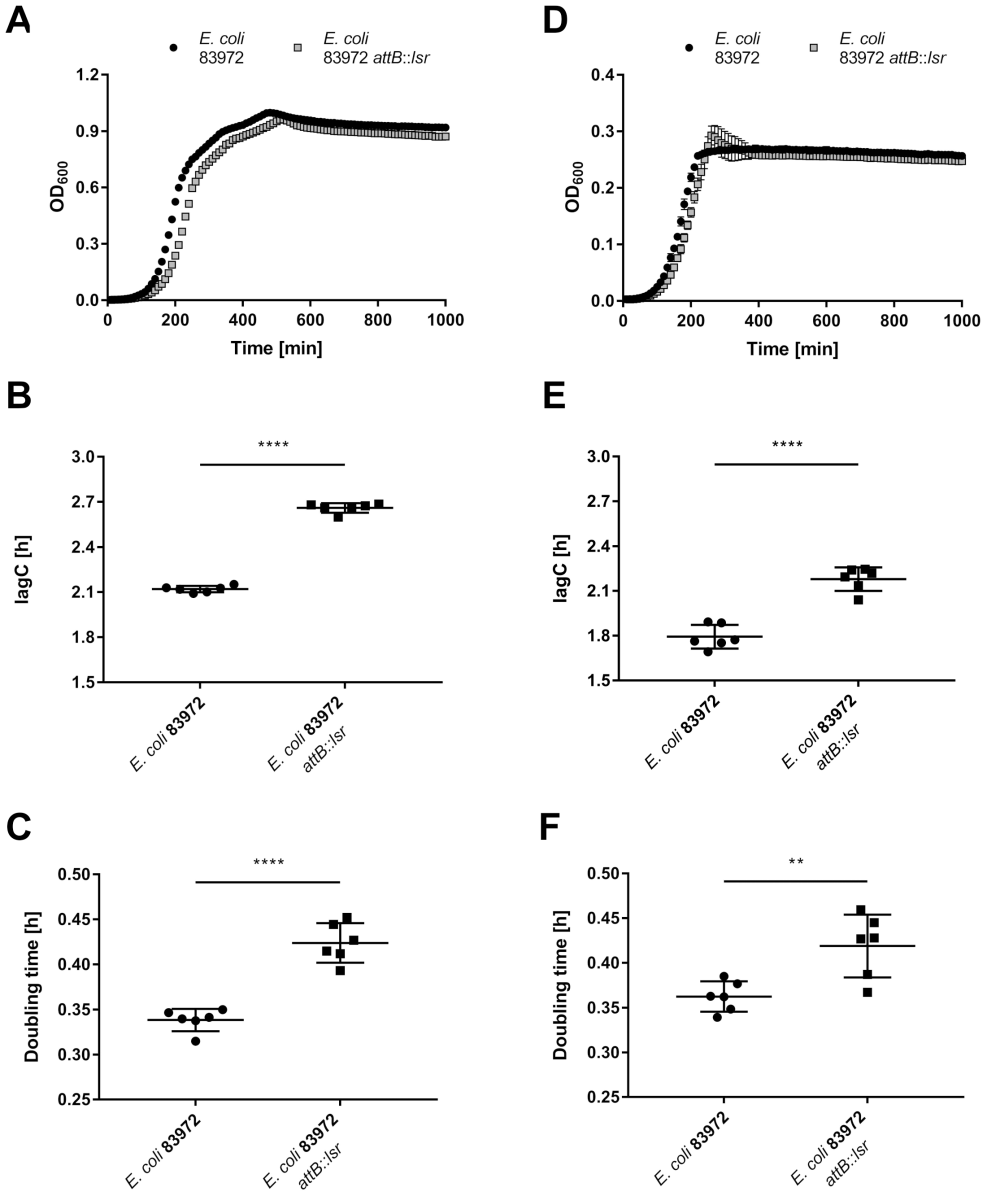
Restoration of the *lsr* determinant impairs growth of *E. coli* 83972. Growth analysis of *E. coli* strains 83972 and 83972 *attB*::*lsr* was done in LB under **(A-C)** aerobic and **(D-F)** anaerobic conditions. **(A, D)** Growth curves over a time span of 1000 min with optical density (OD_600_) measurements every 10 min of *E. coli* 83972 (black circles) and *E. coli* 83972 *attB*::*lsr* (grey squares). **(B, E)** Time until the cultures reached the exponential growth phase (lagC). **(C, F)** Doubling time during the exponential growth phase. Depicted are three biological replicates performed in duplicates each. The starting OD_600_ was 0.01 for both strains. Growth curve analysis was done using AMiGA (Midani et al., 2021). Statistical analysis was performed using an unpaired t-test; values < 0.05 were considered statistically significant (** p<0.01, **** p<0.0001).

**Figure 5:**
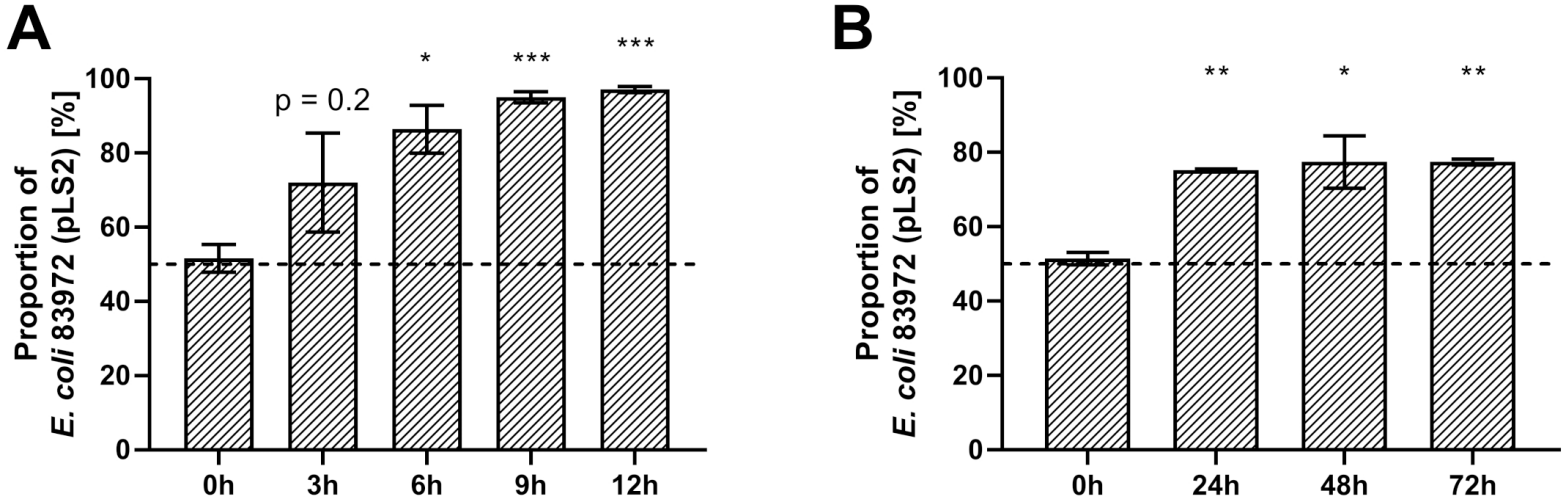
Restoration of the *lsr* determinant reduces competitiveness in serial passaging of *E*. *coli* 83972. Competition assays were done by mixing overnight cultures of strains 83972 (pLS2) and 83972 *attB*::*lsr* (pLS1) in a 1:1 ratio in LB. **(A)** The cultures were grown and subsequently diluted (1:200) into fresh medium every 3 h. Depicted are the mean values and standard deviations of the ratio analysis of ten microscopic pictures for four biological replicates at the indicated time points. **(B)** The cultures were grown over a total of 72 h. Depicted are the mean values and standard deviations of the ratio analysis of ten microscopic pictures for three biological replicates at the indicated time points. Statistical analysis was performed using RM one-way ANOVA (Geisser-Greenhouse correction) with Dunnett’s multiple comparison test; values < 0.05 were considered statistically significant (* p<0.05, ** p<0.01, *** p<0.001).

As the majority of differentially regulated genes affect the bacterial metabolism, we hypothesized that *E. coli* 83972 *attB*::*lsr* might have a growth defect when compared to the wild type strain 83972. Therefore, we analyzed and compared the growth behavior of both strains under aerobic and anaerobic conditions in LB (**Figure 4**) and pooled human urine (**Supplementary Figure S3**). In all conditions tested, *E. coli* 83972 *attB*::*lsr* had a significantly longer lag phase (**Figures 4B, E**; **Supplementary Figures S3B, E**) and a significantly higher doubling time than parental strain 83972 (**Figures 4C, F**; **Supplementary Figures S3C, F**). Next, we performed a growth competition experiment with *E. coli* 83972 and *E. coli* 83972 *attB*::*lsr* to test this phenotype for its biological relevance. To be able to differentiate between both strains by fluorescence microscopy, we introduced the plasmid pLS1 (*cfp* under the control of the stationary phase-dependent *dps* promoter) into *E. coli* 83972 *attB*::*lsr* and the plasmid pLS2 (identical to pLS1 on the nucleotide level, but *cfp* is replaced by *yfp*) into *E. coli* 83972. First, we mimicked bottleneck situations for which we mixed these two strains in a 1:1 ratio and grew the competing strains for 3 h, followed by a subsequent dilution into fresh medium. This cycle was repeated three times. After 3 h of growth, the proportion of *E. coli* 83972 (pLS2) increased from 52 ± 3.7% to 72 ± 13.3% while further increasing to 97 ± 0.8% after three transfers (**Figure 5A**). After 3 h of growth in pooled human urine, the proportion of *E. coli* 83972 (pLS2) rose from 49 ± 6.1% to 61 ± 2.1% while making up 79 ± 1.1% after three serial passages (**Supplementary Figure S4A**). We also tested for the long-term competitiveness of both strains. We observed that the proportion of *E. coli* 83972 (pLS2) significantly rose from 51 ± 1.7% to 75 ± 0.3% in LB after 24 h of direct competition (**Figure 5B**). Within the following 48 h, the proportion of *E. coli* 83972 (pLS2) remained unchanged. In pooled human urine, we saw that the proportion of the wild type strain significantly rose from 48 ± 2.8% to 67 ± 2.7% after 72 h of competition (**Supplementary Figure S4B**).

### 3.4 Reintroduction of *lsr* locus or deletion of *ybhC* leads to a lower resistance against oxidative stress

**Figure 6:**
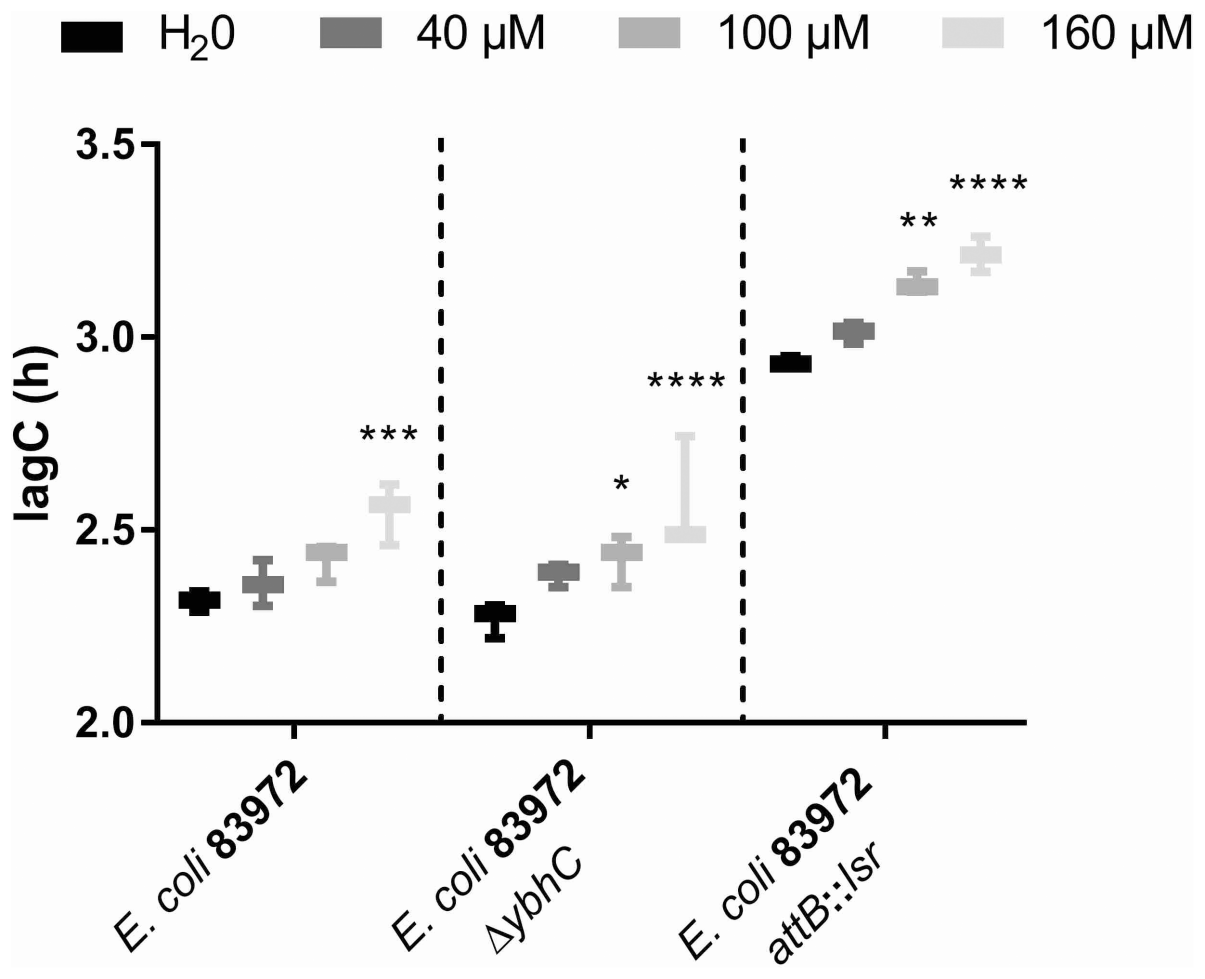
Introduction of the *lsr* locus or deletion of *ybhC* negatively affects oxidative stress resistance of *E. coli* 83972. Shown is the time until the bacteria reach the exponential growth phase (lagC) during growth of *E. coli* strains 83972, 83972 Δ*ybhC* and 83972 *attB*::*lsr* in LB. The starting OD_600_ was 0.01 for each strain. Before growth analysis, the strains were challenged with a final H_2_O_2_ concentration of 40 µM, 100 µM or 160 µM. Water was added as a negative control. LagC was analyzed using AMiGA (Midani et al., 2021). The statistical analysis was performed using ordinary two-way ANOVA with Tukey’s multiple comparison test. Only the effect of different H_2_O_2_ concentrations on the growth of the respective *E. coli* strains was compared (83972, 83972 Δ*ybhC* and 83972 *attB*::*lsr*; simple effect within rows); values < 0.05 were considered statistically significant (** p<0.01, *** p<0.001, **** p<0.0001).

To test for resistance to oxidative stress, we added different concentrations of H_2_O_2_ to low bacterial numbers of the *E. coli* strains 83972, 83972 Δ*ybhC* and 83972 *attB*::*lsr* and analyzed their growth behavior. We observed that the length of the lag phase was significantly longer for *E. coli* 83972 after the addition of 160 µM H_2_O_2_ as compared to the water control, while the addition of 40 µM or 100 µM did not lead to a significantly longer lag phase. In the case of the *E. coli* strains 83972 Δ*ybhC* and 83972 *attB*::*lsr*, on the other hand, already the addition of 100 µM resulted in significantly longer lag phases as compared to the corresponding water controls (**Figure 6**). Once the strains entered exponential growth, however, the doubling times were not affected by the initial H_2_O_2_ stress (**Supplementary Figure S5**).

### 3.5 Complementation of *E. coli* strain 83972 with the *lsr* operon results in impaired colonization capacity in the digestive tract of *Galleria mellonella* larvae

**Figure 7:**
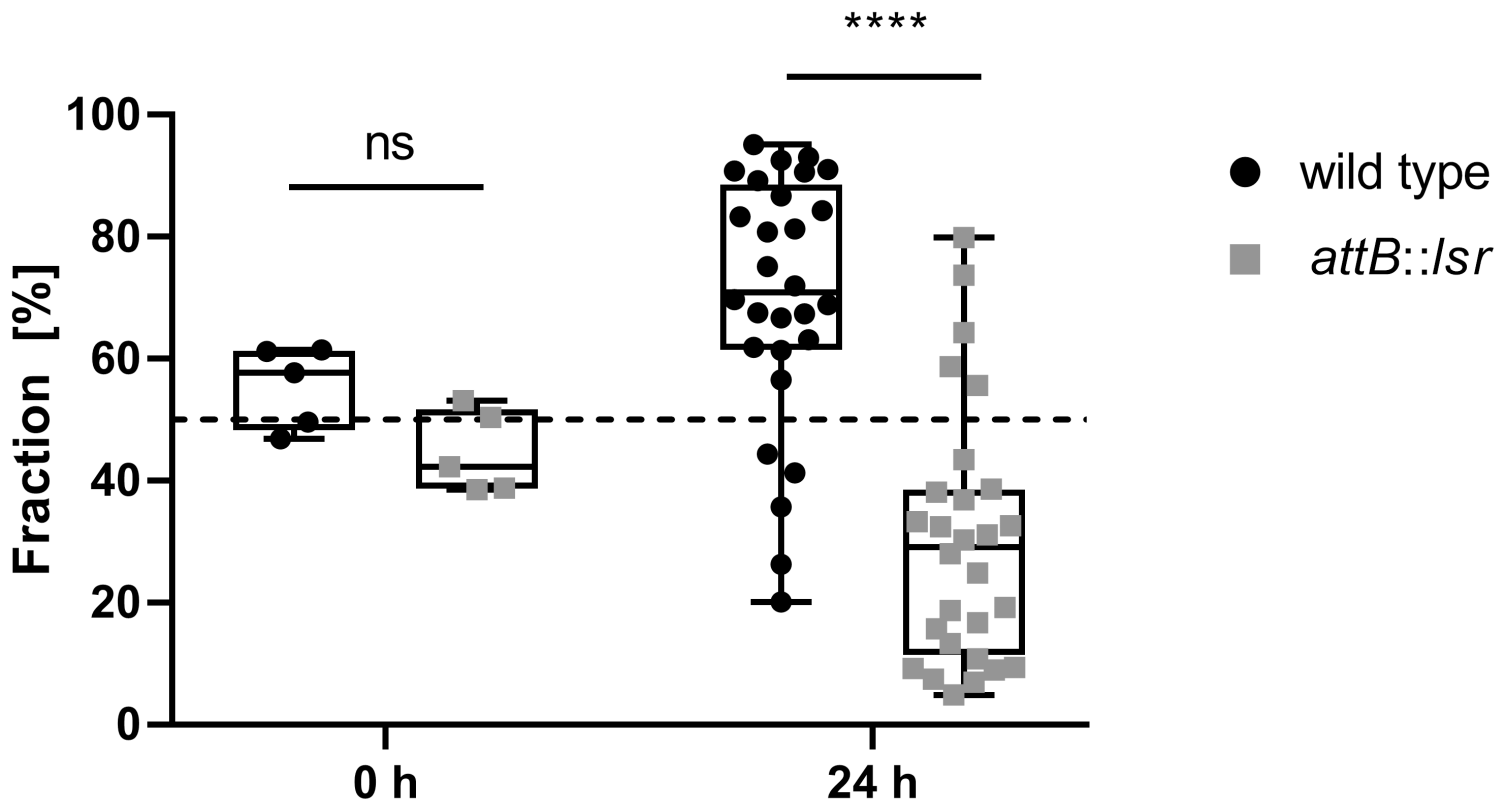
Restoration of the *lsr* locus negatively affects the competitiveness of *E. coli* 83972 in the digestive tract of *Galleria mellonella* larvae. Statistical analysis considering a mixed effects analysis was performed using Sidak’s multiple comparison test; values < 0.05 were considered statistically significant (**** p<0.0001).

Uropathogenic *E. coli* normally have their reservoir in the densely populated intestinal tract, and urinary tract infections usually occur through smear infection with bacteria originating from the intestinal tract. To test whether the presence of a functional *lsr* operon affects the ability of *E. coli* strain 83972, as a degenerate uropathogen, to efficiently colonize a niche that is densely populated by different types of bacteria such as the intestinal tract, we fed *G. mellonella* larvae with a mixture of *E. coli* strain 83972 and its *lsr*-complemented variant in a 1:1 ratio. Quantification after 24 h incubation showed that the *lsr*-positive *E. coli* 83972 variant was found in significantly lower numbers in the digestive tract of the *G. mellonella* larvae than the wild type strain. After 24 hours, the *lsr*-negative wild type *E. coli* 83972 outcompeted its *lsr*-positive complemented counterpart in the digestive tract of *G. mellonella* larvae (**Figure 7**).

### 3.6 *E. coli* of the phylogroup B2 are predominantly *lsr*-negative

**Figure 8:**
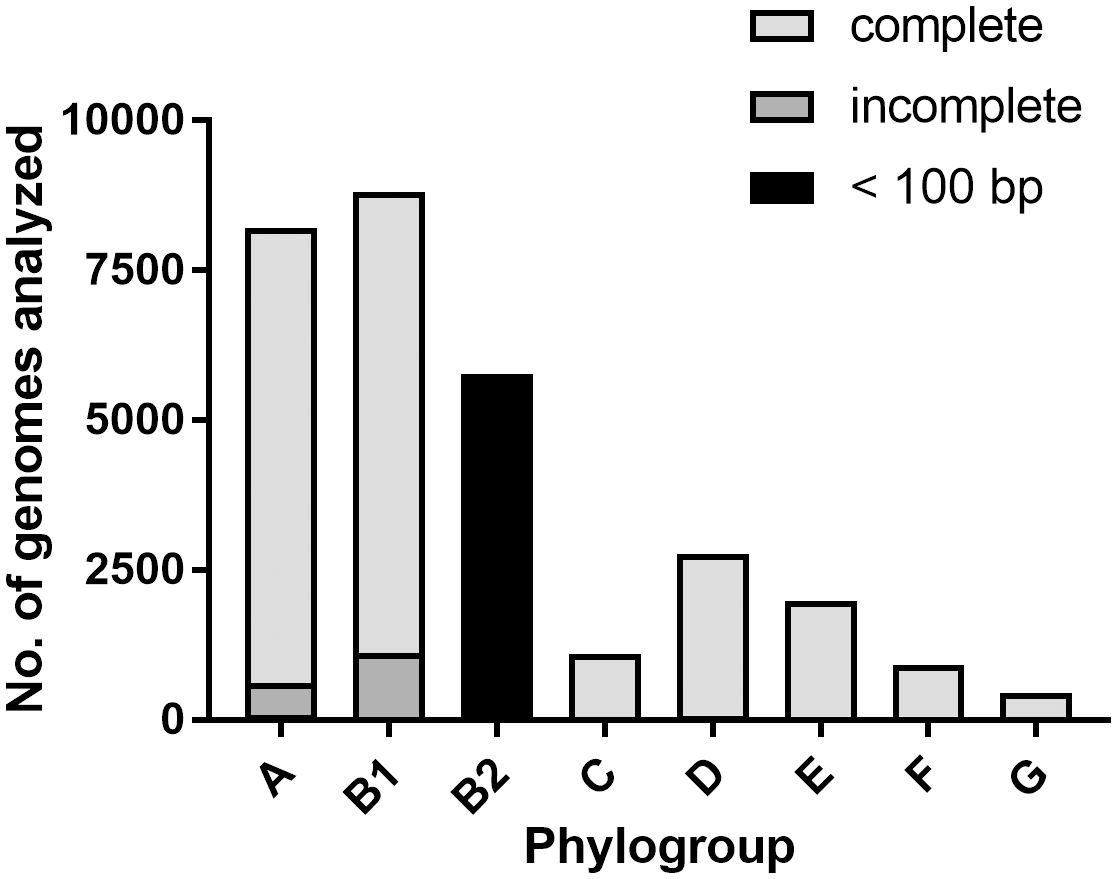
The majority of *E. coli* isolates of the phylogroup B2 does not encode an *lsr* locus. Shown is the distribution of 30,004 *E. coli* genomes with respect to their phylogroup (A, B1, B2, C, D, E, F or G) and the presence of the full-length *lsr* locus (*E. coli* K-12 strain MG1655 *lsrRK-lsrACDBFG*; size: 8,673 bp). “Complete” (light grey) equals a blastn match of 8,673 bp, “incomplete” (dark grey) equals a blastn match < 8,673 bp and “< 100 bp” (black) equals a blastn match < 100 bp.

To obtain an overview of the abundance of the *lsr* locus in the *E. coli* population, we screened all *E. coli* genomes that were available from the NCBI database concerning their phylogroup and the presence of the full-length *lsr* locus. In total, we analyzed 32,404 genomes of isolates from the phylogroups A, B1, B2, C, D, E, F, and G. Genomes from isolates of phylogroup B1 accounted for the largest share (29.1%), followed by phylogroup A (28.2%) and phylogroup B2 (18.6%) (**Supplementary Table S2**). We screened these genomes for the presence of the full-length *lsr* locus (reference DNA sequence from the *E. coli* K-12 strain MG1655) in all genomes. The search resulted in 22,259 complete, 1,861 incomplete and 2,400 multiple matches. In 5,884 genomes, we detected no match or a blastn hit length that was shorter than 100 bp (**Supplementary Table S3**). We found that > 81% of *E. coli* strains belonging to the phylogroups A1, B1, C, D, E, F, or G carry a full-length *lsr* locus. On the contrary, in > 95% of *E. coli* isolates belonging to the phylogroup B2, a homologous region smaller than 100 bp was detected (**Figure 8**, **Supplementary Figure S6A**). We analyzed the prevalence of the *luxS* and *tam* genes in the *E. coli* population. The search for the *luxS* genes in the genome collection resulted in 32,301 complete, 22 incomplete, and 62 multiple matches. In 19 genomes, we found no match (**Supplementary Table S3**). The search for the *tam* gene, which is located downstream of *lsrG* resulted in 30,364 complete, 1,679 incomplete, and 181 multiple matches. In 180 genomes, we found no match (**Supplementary Table S3**). Accordingly, more than 99% of the analyzed *E. coli* genomes include a full-length *luxS* gene (**Supplementary Figure S6B**), and > 88% of the analyzed *E. coli* genomes possess a full-length *tam* gene (**Supplementary Figure S6C**).

## 4 Discussion

AI-2-dependent QS was shown to play an essential role in APEC and MPEC strains for virulence, colonization, or resistance (Palaniyandi et al., 2013; Han et al., 2015; Xue et al., 2016; Zuo et al., 2019; Yu et al., 2020; Wang et al., 2021; Helmy et al., 2022). However, except for knowledge about the role of QS-induced biofilm formation in catheter-associated UTIs (Laganenka and Sourjik, 2018; Henly et al., 2021; Lila et al., 2023), little is known about the function of AI-2-dependent QS in UTIs. *E. coli* 83972 is naturally *lsr*-negative but *luxS*-positive and causes asymptomatic bacteriuria (Medina and Castillo-Pino, 2019). We analyzed this strain’s ability to produce, export, import, and phosphorylate AI-2. We found that *E. coli* 83972 produced AI-2, which could be sensed by another *E. coli* strain during co-culture (**Figure 2B**). The AI-2 production was LuxS-dependent, as judged by the inability to induce AI-2-dependent YFP expression in our reporter strain when *luxS* was deleted in *E. coli* 83972 (**Figure 2B**). However, we observed no AI-2-dependent YFP expression when *E. coli* 83972 Δ*luxS* (pMK2) was challenged with extracellular DPD (**Figures 2D, E**). In comparison, the addition of low concentrations of extracellular DPD to *E. coli* 83972 Δ*luxS* (pMK1) has led to a significantly higher YFP expression compared to the water control (**Figure 2F**). This result was expected since *E. coli* 83972 does not encode LsrK, which is so far the only described kinase that phosphorylates incorporated AI-2 (Pereira et al., 2012). Since AI-2 is initially taken up in an Lsr-independent way by a phosphoenolpyruvate phosphotransferase system, the lack of an active AI-2 kinase would not lead to an AI-2 sequestration inside the cell because unphosphorylated AI-2 is transported back into the extracellular space (Marques et al., 2011; Pereira et al., 2012; Trappetti et al., 2017). When pMK1, a plasmid encoding LsrK, was introduced into *E. coli* 83972, externally added AI-2 was incorporated, phosphorylated, and sequestered inside the cell, while the introduction of pMK2, lacking *lsrK*, did not lead to AI-2 phosphorylation. These results let us conclude that *E. coli* 83972 produces AI-2, which can be sensed by other bacteria but cannot process AI-2 itself. This is not unusual, as other bacteria are also known to be unable to sense AI-2 and express *luxS* only because of its role in the activated methyl cycle (Rezzonico and Duffy, 2008). One example is the uropathogenic *Proteus mirabilis* strain BB2000, which does not possess a *luxP* or *lsrB* homolog but does express *luxS* (Rezzonico and Duffy, 2008). Furthermore, the deletion of *luxS* did not lead to altered colonization or pathogenicity of this strain during UTI in a mouse model (Schneider et al., 2002), suggesting that AI-2-dependent QS does not play a role during UTI in this strain. To reintroduce the ability to use AI-2 as a signaling molecule, we integrated the *lsr* locus into the chromosomal *attB* site of *E. coli* 83972 (**Figure 2A**). Indeed, when *E. coli* 83972 Δ*luxS attB*::*lsr* (pMK2) was challenged with extracellular DPD, we observed a significantly higher YFP expression as compared to the water control (**Figures 2D, E**). This result suggests that *lsrK* is functionally expressed in *E. coli* 83972 *attB*::*lsr*. Additionally, *E. coli* 83972 *attB*::*lsr* could export AI-2, which could be sensed by another *E. coli* strain in coculture (**Figure 2C**).

The observation that global gene expression of UPEC strains *in vivo* during human UTI (hUTI) or murine UTI (mUTI) was more similar to their *in vitro* gene expression in LB than to their *in vitro* gene expression in pooled human urine (Frick-Cheng et al., 2020) allows us to assess the importance of *lsr*-dependent gene expression for colonization of the urinary bladder using our own *in vitro* RNA-seq data in LB. Our transcriptome analysis revealed that the transcriptomes of the *E. coli* strains 83972 and 83972 *attB*::*lsr* were not drastically different during the three growth phases (**Supplementary Figure S2**). Only 60–72 genes were differentially expressed in *E. coli* strains 83972 and 83972 *attB*::*lsr* with a log2-fold change of higher or lower 1 (**Figure 3**). By comparing the results of Frick-Cheng and colleagues (Frick-Cheng et al., 2020) to our data, we observed that some genes or operons that were differentially expressed *in vivo* during hUTI/mUTI relative to *in vitro* growth in LB, also seem to be affected by AI-2-dependent QS. For example, several genes from the flagellar machinery (*flgCFGLM* and *fliS*) were downregulated during mUTI/hUTI as compared to growth in LB (Frick-Cheng et al., 2020). More than 50% of all downregulated genes in *E. coli* 83972 *attB*::*lsr* in the lag phase were linked to the flagellar machinery (**Table 2**). The link between AI-2-dependent QS and flagellar gene expression was also seen in the EHEC strain 86-24, where the deletion of *luxS* led to a downregulation of flagellar genes (Sperandio et al., 2001), which is contradictory to our results where AI-2-dependent QS seems to repress flagellar gene expression. In UPEC strain CFT073, flagella expression was not crucial for bladder colonization efficiency, whereas the loss of flagellar genes was a disadvantage during bladder colonization (Lane et al., 2005). During UTI, the flagella induce a Toll-like receptor-dependent immune response and, thus, inflammation (Subashchandrabose and Mobley, 2015; Acharya et al., 2019). However, since the *flhDC* genes coding for the master regulator of the flagellar biosynthesis cascade (Subashchandrabose and Mobley, 2015), and the highly antigenic flagellin, encoded by *fliC* (Acharya et al., 2019), were not differentially expressed in *E. coli* 83972 *attB*::*lsr* (**Table 2**, **Supplementary Table S1**), and since *E. coli* 83972 expresses only little flagella (Hancock et al., 2008), it appears unlikely that the further downregulation of already weakly expressed flagellar genes is negatively impacting this strain’s bladder colonization ability.

One of the main characteristics of *E. coli* 83972 is its fast growth in urine and, thus, the outgrowth of competing UPEC strains in direct competition (Roos et al., 2006; Ipe et al., 2016). One factor that might contribute to this higher competitiveness is metabolic adaptation (Ipe et al., 2016). We found several genes associated with metabolism to be differentially expressed in all three growth phases in *E. coli* 83972 *attB*::*lsr* (**Table 1**). AI-2-dependent QS was already linked to sugar metabolism (Ha et al., 2018), catabolite repression (Wang et al., 2005) and carbon regulation (Mitra et al., 2016). The strong upregulation of genes involved in the valine, leucine, and isoleucine biosynthesis (*ilv* operon) was interesting since *ilvA* and *ilvC* were found to be essential factors during growth in urine (Vejborg et al., 2012), probably because the overall concentrations of the three amino acids are relatively low as compared to other amino acids in urine (Bouatra et al., 2013) (**Table 1**, **Supplementary Table S1**). In *E. coli* strain CFT073, valine overproduction is considered a metabolic adaptation during biofilm formation (Valle et al., 2008). We also found several genes related to biofilm formation and adhesion upregulated in the transition to the stationary phase upon restoration of the *lsr* operon (**Supplementary Table S1**, **Table 2**). Thus, we checked for biofilm formation according to the protocol of (Laganenka et al., 2016). in *E. coli* strains 83972 and 83972 *attB*::*lsr*, but observed no significant differences (**Supplementary Figure S7**). Thus, the observed *lsr*-dependent upregulation of the *ilv* genes had no marked effect on biofilm formation of *E. coli* strain 83972 *attB*::*lsr*.

We wondered whether the differential regulation of metabolic genes in *E. coli* 83972 *attB*::*lsr* might affect growth. Indeed, we observed a growth retardation of this strain compared to the *lsr*-negative wild type 83972, independent of growth medium or oxygen availability (**Figure 4**, **Supplementary Figure S3**). Consistent with the findings of Sperandio and colleagues made in EHEC strain 86-24 (Sperandio et al., 2001), we observed that the deletion of *luxS* resulted in a significantly longer lag phase of *E. coli* strain 83972 (**Supplementary Figure S8**). Interestingly, the restoration of the *lsr* determinant in *E. coli* 83972 significantly extended the lag phase as well as the doubling time of *E. coli* 83972 (**Supplementary Figure S8**). As we did not observe an additive effect of *luxS* deletion and *lsr* restoration on the length of the lag phase in *E. coli* 83972, and since *luxS* was not differentially expressed in *E. coli* 83972 *attB*::*lsr* in all three growth phases, the longer lag phase of the strains 83972 Δ*luxS* and 83972 Δ*luxS attB*::*lsr* as compared to the wild type might thus be due to the loss of LuxS as metabolic enzyme (Schauder et al., 2001; Vendeville et al., 2005; Zdziarski et al., 2008) and not due to AI-2 secretion, as *E. coli* 83972 and *E. coli* 83972 Δ*luxS* are not able to sense AI-2. Taken together, these findings showed that the presence of the full-length *lsr* locus alone was not sufficient to cause the observed slowdown in growth but that it most likely resulted from an AI-2-dependent deregulation of specific gene expression. Because we performed the complementation as a single copy-insertion of the *lsr* determinant into a neutral site of the chromosome, overexpression effects and artificial phenotypes due to incompatibilities in gene regulation or metabolic stress caused by overexpression should be excluded. To validate our assumption, additional *E. coli* isolates will have to be tested for their *lsr*-dependent growth characteristics. In this context, fitness costs from the two *cat* and *aph* resistance cassettes used for complementation could be considered as well as non-QS-related functions of the *lsr* determinant.

In the intestinal tract, where the bacterial composition is diverse and the bacterial load is high, a negative effect of AI-2-dependent QS on growth rate does not necessarily lead to a reduction in fitness as AI-2-dependent QS significantly impacts microbiota composition, which in turn affects the fitness of individual bacterial species (Christiaen et al., 2014; Thompson et al., 2015; Deng et al., 2022). Nevertheless, our feeding experiments of *G. mellonella* larvae with equal ratios of *E. coli* 83972 wild type and the *lsr*-complemented strain impressively showed that the restoration of the *lsr* operon significantly reduced the colonization ability and competitiveness of *E. coli* 83972 *attB*::*lsr* in the digestive tract compared to the *lsr*-negative parent strain. We are fully aware that the human intestinal tract and that of *G. mellonella* larvae differ substantially, not only in their microbiome composition. We applied *G. mellonella* larvae as a 3R-compliant model to investigate the ability of *E. coli* 83972 to assert itself in a niche densely populated by a complex microbiota. We view this experiment as a proxy for intestinal colonization and understand that a more relevant colonization model for the human digestive tract must be used in further studies to clarify the impact of AI-2-dependent QS on the intestinal colonization capacity of phylogroup B2 strains. It has recently been described in a mouse model that AI-2 production increases chemotaxis and thus the metabolic trait-dependent fitness of *E. coli* strain Z1331 in the intestine. AI-2-dependent chemotaxis can also promote the coexistence of different *E. coli* strains in the intestine through niche segregation (Laganenka et al., 2023). Related to hardly flagellated *E. coli* strain 83972, we interpret our results as indicating that the importance of AI-2 in bacterial fitness in the digestive tract can be strain-dependent and influenced by individual metabolic and phenotypic characteristics. In the human bladder, however, where bacterial numbers are typically not as high as in the intestinal tract (Perez-Carrasco et al., 2021), regulating growth by monitoring cell density may be a disadvantage for urine isolates, because of rapid changes of the population size in the bladder. The urine void drastically reduces the overall bacterial count and volume of the growth compartment, urging non-voided bacteria to regrow fast to maintain bladder colonization, especially when competing with a second bacterial species (Ipe et al., 2016). QS is known to slow down growth and metabolism as soon as a high cell density is reached (Sperandio et al., 2001; An et al., 2014). When the bacteria encounter a rapid reduction in cell density, this switch leads to a subsequent adaptation of gene expression in quorum sensing strains (Pereira et al., 2013), which likely differs from that in bacteria that cannot sense the bacterial cell density. In our co-cultivation experiment, where the *lsr*-positive strain 83972 *attB*::*lsr* (pLS1) and the *lsr*-negative strain 83972 (pLS2) were in direct competition, the *lsr*-positive variant capable of QS was outcompeted by the isogenic *lsr*-negative wild type, which is unable to sense bacterial cell density but displays a shorter lag phase and doubling time (**Figure 4**). The observed slowdown in growth and prolonged doubling time of the *lsr*-positive variant could underlie the displacement of this mutant by the QS-insensitive wild type due to QS-dependent adaptations in gene expression. Especially in the case of frequent changes in population density, here mimicked by serial passage after 3 h of growth (**Figure 5A**), compared to the long-term competition experiment (**Figure 5B**), the displacement by the *lsr*-negative strain is particularly evident. This property benefits *E. coli* 83972 in bacterial interference in the urinary bladder because it can compensate for bottlenecks due to rapid reductions in population size as a result of repeated voiding. In summary, our results suggest that the differential expression of metabolic genes by AI-2-dependent QS may result in different growth characteristics and reduced competitiveness of *E. coli* 83972 *attB*::*lsr*. The direct evidence, however, is still missing, and the precise regulatory mechanisms remain to be elucidated.

The *nrdHIEF* operon was upregulated during mUTI/hUTI as compared to growth in LB (Frick-Cheng et al., 2020), and we found *nrdHIEF* upregulated in *E. coli* 83972 *attB*::*lsr* as compared to *E. coli* 83972 in the lag phase (**Table 3**). Though the operon encodes a ribonucleotide reductase that is required for dNTP synthesis and is thus associated with DNA synthesis, *nrdHIEF* expression was found to be stimulated upon oxidative stress, particularly in mutants that miss major antioxidant defenses (Monje-Casas et al., 2001). It is thought that *E. coli* overexpresses several reductases and electron donors, including *nrdHIEF*, to cope with oxidative stress (Monje-Casas et al., 2001; Abdelwahed et al., 2022). Interestingly, the gene *ybhC*, which leads to a significantly lower minimal inhibitory concentration against H_2_O_2_ when deleted in the *E. coli* strain BW25113 (Chen et al., 2021), was the most strongly downregulated gene during the lag and exponential growth phase and was also strongly downregulated during the transition to the stationary phase in *E. coli* 83972 *attB*::*lsr* (**Supplementary Table S1**). We wondered whether this strong downregulation might contribute to a higher sensitivity to H_2_O_2_. Therefore, we challenged *E. coli* 83972, its isogenic *ybhC* deletion mutant 83972 Δ*ybhC*, and *E. coli* 83972 *attB*::*lsr* with H_2_O_2_ concentrations that can be physiologically measured in human urine (Varma and Devamanoharan, 1990). Indeed, we observed that *E. coli* 83972 was more resistant to H_2_O_2_ than the strains 83972 Δ*ybhC* and 83972 *attB*::*lsr* (**Figure 6**). We observed that the *ybhC* deletion did not affect the length of the lag phase as compared to *E. coli* 83972 (unpaired t-test; p = 0.19), whereas *E. coli* 83972 *attB*::*lsr* had a significantly longer lag phase than both, *E. coli* 83972 and *E. coli* 83972 Δ*ybhC* (**Figure 6**). Thus, the overall longer lag phase of the *lsr*-positive variant of strain 83972 does not explain its higher sensitivity to H_2_O_2_, while the strong downregulation of *ybhC* in this strain might be one factor responsible for this phenotype. Notably, the increased H_2_O_2_ sensitivity of *E. coli* 83972 *attB*::*lsr* is in agreement with a previous report of increased H_2_O_2_ sensitivity in the MPEC strain DCM5 expressing a functional LsrR (Wang et al., 2021). However, in this MPEC strain, the higher sensitivity was explained by LsrR-mediated repression of H_2_O_2_ scavenging enzyme-encoding genes, including *ahpCF*. As opposed to that, we found that *ahpCF* was slightly upregulated in strain 83972 *attB*::*lsr* in the lag phase (**Supplementary Table S1**). Therefore, the decreased expression of *ahpCF* is unlikely to explain the *lsr*-dependent sensitivity of *E. coli* 83972 to H_2_O_2_. *E. coli* 83972 has an increased level of endogenous reactive oxygen species while growing in urine compared to other ABU and UPEC strains, but correspondingly, it also has a more active antioxidant defense system (Aubron et al., 2012). We found several differentially expressed genes in *E. coli* 83972 *attB*::*lsr* that have been associated with oxidative stress in other studies (**Supplementary Table S4**) or other stress-related responses (**Table 4**). Thus, the overall H_2_O_2_ detoxification or stress response seems to be imbalanced in the presence of functional AI-2-dependent QS in *E. coli* 83972 *attB*::*lsr*, resulting in a higher sensitivity against oxidative stress. During ABU, however, increased sensitivity to H_2_O_2_ is a disadvantage since the infiltration of activated neutrophils into the bladder represents one host defense mechanism to kill bacteria with reactive oxygen species during UTI (Demirel et al., 2020). Especially in a competition situation in the bladder, a significantly prolonged lag phase and doubling time of *E. coli* 83972 *attB*::*lsr* in addition to the increased H_2_O_2_ sensitivity could constitute a clear colonization disadvantage relative to the *lsr*-negative wild type strain (**Supplementary Figure S5**).

AI-2-dependent QS in ExPEC has pleiotropic effects since it contributes to virulence, colonization, and antibiotic resistance but also H_2_O_2_ sensitivity, metabolism, and growth defects, as can also be seen in our study (Xue et al., 2009; Kathayat et al., 2021; Wang et al., 2021). Epidemiological studies have shown that ExPEC frequently belong to the phylogroup B2 (Picard et al., 1999; Micenková et al., 2016). However, in the collection of *E. coli* genomes we examined, genomes of phylogroup A and B1 isolates were predominant (**Supplementary Table S2**). Intriguingly, it was already suggested that *E. coli* strains of the phylogroup B2 might have lost the *lsr* locus during evolution (Quan and Bentley, 2012; Brito et al., 2013). However, all B2 strains analyzed by Brito and colleagues encoded a functional LuxS synthase and thus should be capable of producing AI-2 (Brito et al., 2013). Following Brito and colleagues, we found that the vast majority of *E. coli* belonging to the phylogroup B2 lack a full-length *lsr* locus (**Figure 8**, **Supplementary Figure S6A**), while the presence of *luxS* seems to be remarkably conserved (**Supplementary Figure S6B**). The high prevalence of *luxS* in *E. coli* and also over a long time in bacteria (Santiago-Rodriguez et al., 2014) underlines its crucial role as a metabolic enzyme in the activated methyl cycle (Vendeville et al., 2005). The *tam* gene, which is located downstream of the *lsrACDBFG* operon, was also widely distributed (> 88%) in the different phylogroups (**Supplementary Figure S6C**). Since *E. coli* 83972 encodes Tam and its role in AI-2-dependent QS remains unclear, we omitted *tam* from the complemented *lsr* locus. Indeed, we observed that *tam* was upregulated during the transition to the stationary phase (data not shown) but was not differentially expressed in *E. coli* 83972 *attB*::*lsr* as compared to *E. coli* 83972. Evolutionary studies suggest that the *lsr* locus was passed on via horizontal gene transfer (Rezzonico et al., 2012), and lateral gene transfer events were also described (Pereira et al., 2009). Thus, it is interesting that the operon was not reintroduced into the phylogroup B2 (Manges and Johnson, 2015). These observations and our data suggest that the loss of AI-2-dependent QS could be an evolutionary advantage for *E. coli* 83972, which efficiently colonizes the urinary bladder as an extraintestinal body niche. Whether the absence of the *lsr* determinant in almost all phylogroup B2 isolates investigated so far may be correlated, at least in part, with generally increased fitness properties in extraintestinal niches or superior colonization properties in densely populated niches such as the intestinal tract will require more detailed future analyses of the effect of *lsr* complementation in a diverse collection of phylogroup B2 strains.

## 5 Conclusion

It is imperative that we explore non-antibiotic solutions to prevent UTI, with bacterial interference being a promising avenue for treating symptomatic episodes of uncomplicated cystitis. Our study provides new information on gene regulation in *E. coli* 83972. The more we understand which bacterial characteristics improve fitness and colonization properties in the urinary bladder, the better we could rationally improve the engineering of *E. coli* 83972 or predict outcomes in therapeutic colonization. The impact of AI-2-mediated QS for pathogenicity or fitness of ExPEC is not uniform and is probably dependent on the individual strain background. Whereas interference with AI-2-dependent QS resulted in downregulation of ExPEC virulence traits and attenuation in some isolates, increased fitness due to enhanced resistance to oxidative stress was observed in others. It is tempting to speculate that the absence of the *lsr* operon in the majority of phylogroup B2 isolates may potentially suggest that the loss of AI-2-dependent QS could be associated with a fitness advantage in extraintestinal niches or their reservoir, i.e., the intestinal tract. However, this must be further analyzed in detail in the future. In this study, we investigated the impact of AI-2-mediated QS on fitness traits of asymptomatic bacteriuria *E. coli* isolate 83972, a strain that has already been used for therapeutic bladder colonization, thereby interfering with bladder infections caused by uropathogens. We found that the reintroduction of AI-2-dependent QS in *E. coli* 83972, which is optimally adapted to the growth conditions in the urinary bladder, has led to a phenotype that is disadvantageous for efficient and long-term bladder colonization, especially in a competitive situation. Restoration of the *lsr* determinant in *E. coli* 83972 resulted in growth retardation, loss of competitiveness and increased susceptibility to oxidative stress, all characteristics relevant to this strain’s colonization ability in the bladder. Thus, our findings indicate that the absence of AI-2-dependent QS in *E. coli* 83972 may be an advantage during the colonization of the urinary bladder. Further studies on the benefits of the lack of the *lsr* determinant in a broad spectrum of ExPEC isolates with different phylogenetic backgrounds and genome content are needed to gain deeper insights into the general importance of AI-2-dependent QS for *E. coli* fitness and pathogenicity in extraintestinal niches and or the colonization of the intestinal tract.

## Data Availability

The datasets presented in this study are publicly accessible at NCBI GEO (SRA accession number GSE300954). This data can be found here: (https://www.ncbi.nlm.nih.gov/geo/query/acc.cgi?acc=GSE300954).
