# Supplementary material for: Less is more: the lack of autoinducer-2-dependent quorum sensing promotes competitive fitness of Escherichia coli strain 83972
Source: Front Cell Infect Microbiol. 2025 Jul 2;15:1603759. doi: 10.3389/fcimb.2025.1603759 (PMC12263705; doi:10.3389/fcimb.2025.1603759)
Supplement: Supplementary file 2 [file Table2.docx]

Supplementary Material

**Supplementary Table 2: Phylogroup distribution of analysed *E*. *coli* strains.** Shown is the distribution of 32,404 *E*. *coli* genomes with respect to their phylogroups. Genomes were downloaded from NCBI Assembly ([Kitts et al., 2016](#_ENREF_18)), and the *E*. *coli* phylogroup affiliation was determined using ClermonTyping ([Beghain et al., 2018](#_ENREF_6)).

| Phylogroup | Number of genomes | Percentage |
| --- | --- | --- |
| A | 9133 | 28.2 |
| B1 | 9415 | 29.1 |
| B2 | 6022 | 18.6 |
| C | 1169 | 3.6 |
| D | 3093 | 9.5 |
| E | 2072 | 6.4 |
| F | 1011 | 3.1 |
| G | 489 | 1.5 |

**Supplementary Table 3: Conservation of the *lsr* locus, *luxS* and *tam* according to the phylogroup.**

| Gene name(s) |  | BLAST match | | | | Percentages | | | |
| --- | --- | --- | --- | --- | --- | --- | --- | --- | --- |
| *lsr* locus (*lsrRK*-*lsrACDBFG*) |  | Equal 8673 bp | Unequal 8673 bp | < 100 bp | Multiple matches | Equal 8673 bp | Unequal 8673 bp | < 100 bp | Multiple matches |
|  | A | 7593 | 537 | 81 | 922 | 83.14 | 5.88 | 0.89 | 10.1 |
|  | B1 | 7707 | 1085 | 24 | 599 | 81.86 | 11.52 | 0.25 | 6.36 |
|  | B2 | 11 | 15 | 5751 | 245 | 0.18 | 0.25 | 95.5 | 4.07 |
|  | C | 1069 | 19 | 5 | 76 | 91.45 | 1.63 | 0.43 | 6.5 |
|  | D | 2692 | 62 | 11 | 328 | 87.04 | 2 | 0.36 | 10.6 |
|  | E | 1924 | 55 | 0 | 93 | 92.86 | 2.65 | 0 | 4.49 |
|  | F | 866 | 43 | 12 | 90 | 85.66 | 4.25 | 1.19 | 8.9 |
|  | G | 397 | 45 | 0 | 47 | 81.19 | 9.2 | 0 | 9.61 |
| *luxS* |  | Equal 516 bp | Unequal 516 bp | No match | Multiple matches | Equal 516 bp | Unequal 516 bp | No match | Multiple matches |
|  | A | 9108 | 8 | 5 | 12 | 99.73 | 0.09 | 0.05 | 0.13 |
|  | B1 | 9401 | 0 | 5 | 9 | 99.85 | 0 | 0.05 | 0.1 |
|  | B2 | 5980 | 12 | 5 | 25 | 99.3 | 0.2 | 0.08 | 0.42 |
|  | C | 1167 | 0 | 0 | 2 | 99.83 | 0 | 0 | 0.17 |
|  | D | 3082 | 1 | 2 | 8 | 99.64 | 0.03 | 0.06 | 0.26 |
|  | E | 2066 | 1 | 2 | 3 | 99.71 | 0.05 | 0.1 | 0.14 |
|  | F | 1009 | 0 | 0 | 2 | 99.8 | 0 | 0 | 0.2 |
|  | G | 488 | 0 | 0 | 1 | 99.8 | 0 | 0 | 0.2 |
| *tam* |  | Equal 759 bp | Unequal 759 bp | No match | Multiple matches | Equal 759 bp | Unequal 759 bp | No match | Multiple matches |
|  | A | 8921 | 104 | 78 | 30 | 97.68 | 1.14 | 0.85 | 0.33 |
|  | B1 | 8519 | 841 | 23 | 32 | 90.48 | 8.93 | 0.24 | 0.34 |
|  | B2 | 5336 | 524 | 62 | 100 | 88.61 | 8.7 | 1.03 | 1.66 |
|  | C | 1156 | 2 | 6 | 5 | 98.89 | 0.17 | 0.51 | 0.43 |
|  | D | 3073 | 11 | 3 | 6 | 99.35 | 0.36 | 0.1 | 0.19 |
|  | E | 1880 | 188 | 1 | 3 | 90.73 | 9.07 | 0.05 | 0.14 |
|  | F | 994 | 8 | 7 | 2 | 98.32 | 0.79 | 0.69 | 0.2 |
|  | G | 485 | 1 | 0 | 3 | 99.18 | 0.2 | 0 | 0.61 |

**Supplementary Table 4: Differentially expressed genes in *E*. *coli* 83972 *attB*::*lsr* associated with oxidative stress.**

|  | **Gene name** | **log2-fold change** | **Pathway/Function** ([Kanehisa and Goto, 2000](#_ENREF_16);[Kanehisa, 2019](#_ENREF_14);[Kanehisa et al., 2023](#_ENREF_15)) | **Reference** |
| --- | --- | --- | --- | --- |
| **lag phase** |  |  |  |  |
|  | *ybhC* | -3.44271322 | Pectinesterase; Putative lipoprotein | ([Chen et al., 2021](#_ENREF_10)) |
|  | *ssuD* | -1.7364083 | Alkanesulfonate Monooxygenase; Sulphur metabolism | ([Roth et al., 2022](#_ENREF_29)) |
|  | *yhfG* | -1.40912476 | Conserved hypothetical protein | ([Abdelwahed et al., 2022](#_ENREF_2)) |
|  | *flgE* | -1.19994955 | Flagellar hook protein; Flagellar assembly | ([Chen et al., 2021](#_ENREF_10)) |
|  | *ycjF* | -1.10548474 | Putative inner membrane protein | ([Abdelwahed et al., 2022](#_ENREF_2)) |
|  | *cpxP* | -1.01263736 | Chaperone; Periplasmic protein | ([Lopez et al., 2018](#_ENREF_21)) |
|  | *cadA* | -0.92972157 | Lysine decarboxylase; Lysine degradation | ([Abdelwahed et al., 2022](#_ENREF_2)) |
|  | *ygaC* | -0.88023273 | Conserved hypothetical protein | ([Abdelwahed et al., 2022](#_ENREF_2)) |
|  | *rbsA* | -0.80719956 | ABC-type D-ribose transporter | ([Chen et al., 2021](#_ENREF_10)) |
|  | *yheL* | -0.62753819 | Putative intracellular sulphur oxidation protein; tRNA biogenesis | ([Abdelwahed et al., 2022](#_ENREF_2)) |
|  | *ibpB* | -0.57727775 | Heat shock protein | ([Zheng et al., 2001](#_ENREF_34)) |
|  | *ibpA* | -0.49969182 | Small heat shock protein | ([Zheng et al., 2001](#_ENREF_34)) |
|  | *rpmG* | 0.29762026 | Large subunit ribosomal protein; L33 | ([Chen et al., 2021](#_ENREF_10)) |
|  | *ahpC* | 0.36821649 | Alkyl hydroperoxide reductase subunit C; NADH-dependent peroxiredoxin | ([Seaver and Imlay, 2001](#_ENREF_31)); ([Chen et al., 2021](#_ENREF_10)) |
|  | *ahpF* | 0.41356442 | Alkyl hydroperoxide reductase subunit F | ([Seaver and Imlay, 2001](#_ENREF_31)); ([Chen et al., 2021](#_ENREF_10)) |
|  | *rho* | 0.45274385 | Transcription termination factor; RNA degradation | ([Chen et al., 2021](#_ENREF_10)) |
|  | *upp* | 0.50345781 | Uracil phosphoribosyltransferase; Pyrimidine metabolism | ([Chen et al., 2021](#_ENREF_10)) |
|  | *tpx* | 0.55321827 | Thioredoxin-dependent peroxiredoxin; Thiol peroxireductase | ([Chen et al., 2021](#_ENREF_10)) |
|  | *sodB* | 0.58247572 | Superoxide dismutase | ([Chen et al., 2021](#_ENREF_10)) |
|  | *nrdF* | 1.0340548 | Ribonucleoside-diphosphate reductase; Deoxyribonucleotide biosynthesis | ([Monje-Casas et al., 2001](#_ENREF_25)); ([Abdelwahed et al., 2022](#_ENREF_2)) |
|  | *nrdH* | 1.13985791 | Glutaredoxin-like protein; Deoxyribonucleotide biosynthesis | ([Monje-Casas et al., 2001](#_ENREF_25)); ([Abdelwahed et al., 2022](#_ENREF_2)) |
|  | *nrdE* | 1.2692191 | Ribonucleoside-diphosphate reductase; Deoxyribonucleotide biosynthesis | ([Monje-Casas et al., 2001](#_ENREF_25)); ([Abdelwahed et al., 2022](#_ENREF_2)) |
|  | *nrdI* | 1.30323686 | Flavodoxin; Deoxyribonucleotide biosynthesis | ([Monje-Casas et al., 2001](#_ENREF_25)); ([Abdelwahed et al., 2022](#_ENREF_2)) |
|  | *ilvA* | 2.26260477 | Threonine ammonia-lyase; Valine, leucine and isoleucine biosynthesis | ([Chen et al., 2021](#_ENREF_10)) |
|  | *ilvM* | 2.34531415 | Acetolactate synthase; Valine, leucine and isoleucine biosynthesis | ([Abdelwahed et al., 2022](#_ENREF_2)) |
| **exp. phase** |  |  |  |  |
|  | *ybhC* | -3.00565158 | Pectinesterase; Putative lipoprotein | ([Chen et al., 2021](#_ENREF_10)) |
|  | *fhuF* | -2.01302934 | Ferric iron reductase | ([Roth et al., 2022](#_ENREF_29)) |
|  | *ycdO* | -1.35459265 | Electrochemical potential-driven transporter; Iron uptake | ([Chen et al., 2021](#_ENREF_10)) |
|  | *sodA* | -1.13285429 | Superoxide dismutase | ([Gao et al., 2019](#_ENREF_13)) |
|  | *soxS* | -0.97051074 | Transcription factor; Regulation of superoxide response regulon | ([Zheng et al., 2001](#_ENREF_34)); ([Roth et al., 2022](#_ENREF_29)) |
|  | *sucA* | 0.82941826 | 2-oxoglutarate dehydrogenase E1 component; Citrate cycle | ([Chen et al., 2021](#_ENREF_10)) |
|  | *ibpA* | 0.97099026 | Small heat shock protein | ([Zheng et al., 2001](#_ENREF_34)) |
|  | *bssS* | 1.02819806 | Type II TA-system protein; Biofilm regulator | ([Abdelwahed et al., 2022](#_ENREF_2)) |
|  | *inaA* | 1.25401735 | pH-inducible protein involved in stress response | ([Manchado et al., 2000](#_ENREF_23)) |
|  | *dsdA* | 1.26593119 | D-serine ammonia-lyase; Amino acid metabolism | ([Zheng et al., 2001](#_ENREF_34)) |
|  | *sucC* | 1.27145525 | Succinyl-CoA synthetase beta subunit; Citrate cycle | ([Chen et al., 2021](#_ENREF_10)) |
|  | *ilvA* | 1.78432476 | Threonine ammonia-lyase; Valine, leucine and isoleucine biosynthesis | ([Chen et al., 2021](#_ENREF_10)) |
|  | *ibpB* | 2.07702689 | Heat shock protein | ([Zheng et al., 2001](#_ENREF_34)) |
| **stat. phase** |  |  |  |  |
|  | *thrA* | -1.85634241 | Aspartate kinase; Amino acid metabolism | ([Chen et al., 2021](#_ENREF_10)) |
|  | *ybhC* | -1.56716214 | Pectinesterase; Putative lipoprotein | ([Chen et al., 2021](#_ENREF_10)) |
|  | *ssuB* | -1.4137389 | Sulfonate transporter; Sulphur metabolism | ([Roth et al., 2022](#_ENREF_29)) |
|  | *lacZ* | -1.01592483 | Beta-galactosidase; Galactose metabolism | ([Chen et al., 2021](#_ENREF_10)) |
|  | *yecI* | -0.85670724 | Putative ferritin-like protein | ([Chen et al., 2021](#_ENREF_10)) |
|  | *zntA* | -0.82564063 | P-type zinc/cadmium transporter | ([Chen et al., 2021](#_ENREF_10)) |
|  | *yaiE* | -0.68333525 | Nucleoside phosphorylase; Nucleotide metabolism | ([Chen et al., 2021](#_ENREF_10)) |
|  | *katG* | -0.60752933 | Catalase-peroxidase; HPI | ([Chen et al., 2021](#_ENREF_10)) |
|  | *yjfN* | -0.58299457 | Hypothetical protein | ([Chen et al., 2021](#_ENREF_10)) |
|  | *yidC* | -0.51252373 | Inner membrane protein insertase | ([Chen et al., 2021](#_ENREF_10)) |
|  | *hokD* | 0.45441772 | Homologue of hok; Small toxic polypeptide | ([Chen et al., 2021](#_ENREF_10)) |
|  | *slyB* | 0.51893239 | Outer membrane lipoprotein | ([Chen et al., 2021](#_ENREF_10)) |
|  | *ycaC* | 0.65166403 | Isochorismatase family protein | ([Abdelwahed et al., 2022](#_ENREF_2)) |
|  | *katE* | 0.67291872 | Catalase; HPII | ([Chen et al., 2021](#_ENREF_10)) |
|  | *ycgZ* | 0.8344795 | Probable RcsB/C two-component-system connector | ([Abdelwahed et al., 2022](#_ENREF_2)); ([Zheng et al., 2001](#_ENREF_34)) |
|  | *upp* | 0.89562746 | Uracil phosphoribosyltransferase; Pyrimidine metabolism | ([Chen et al., 2021](#_ENREF_10)) |
|  | *ytfK* | 0.92556378 | Conserved hypothetical protein | ([Chiang and Schellhorn, 2012](#_ENREF_11)) |
|  | *ybgS* | 0.98346783 | Putative homeobox protein | ([Abdelwahed et al., 2022](#_ENREF_2)) |
|  | *yrbL* | 1.01071298 | Conserved hypothetical protein | ([Chen et al., 2021](#_ENREF_10)) |
|  | *ilvA* | 1.07418499 | Threonine ammonia-lyase; Valine, leucine and isoleucine biosynthesis | ([Chen et al., 2021](#_ENREF_10)) |
|  | *aceK* | 1.19206076 | Isocitrate dehydrogenase kinase/phosphatase | ([Chen et al., 2021](#_ENREF_10)) |
|  | *ycfR* | 1.26232563 | Type II TA-system protein; Multiple stress resistance protein | ([Zheng et al., 2001](#_ENREF_34)); ([Roth et al., 2022](#_ENREF_29)) |

**Supplementary Table 5: Bacterial strains used in this study.**

| Bacterial strain | Description | Abbreviation in the text | Reference |
| --- | --- | --- | --- |
| *E*. *coli* K-12 strain MG1655 | Template for full-length *lsr* locus |  | ([Bachmann, 1972](#_ENREF_4)) |
| *E*. *coli* strain MG1655 *lsrA-G*::*yfp-cat* | *lsrACDBFG* substitution with *yfp* open reading frame coupled to chloramphenicol resistance cassette *cat*; template for the *lsrR:lsrA*-promoter-*yfp* fusion for pMK2 |  | ([Keizers et al., 2022](#_ENREF_17)) |
| *E*. *coli* strain MG1655 (pKD46) | *E*. *coli* K-12 strain MG1655; harbouring the plasmid pKD46 for Red/ET recombineering ([Datsenko and Wanner, 2000](#_ENREF_12)) |  | This study |
| *E*. *coli* strain MG1655 *cat*_*lsr* | *E*. *coli* K-12 strain MG1655; *cat* introduced upstream of the *lsr* locus |  | This study |
| *E*. *coli* K-12 MG1655 *cat*_*lsr* (pKD46) | *E*. *coli* K-12 strain MG1655; *cat* introduced upstream of the *lsr* locus; harbouring the plasmid pKD46 for Red/ET recombineering ([Datsenko and Wanner, 2000](#_ENREF_12)) |  | This study |
| *E*. *coli* strain MG1655 *cat*_*lsr_aph* | *E*. *coli* K-12 strain MG1655; *cat* introduced upstream and *aph* introduced downstream of the *lsr* locus |  | This study |
| *E*. *coli* strain MG1655 Δ*luxS* *attB*::P*lsrA-yfp* | *luxS*::*Sh ble*; *lsrRK:lsrA*-promoter-*yfp* fusion integrated into the λ phage *attB* integration-site (reviewed in ([Campbell, 1992](#_ENREF_9))) | *E*. *coli* Δ*luxS* (*attB*::P*lsrA*-*yfp*) | ([Keizers et al., 2022](#_ENREF_17)) |
| *E*. *coli* strain DH5α (pWKS30_LSR) | Lab strain harbouring the pWKS30_LSR plasmid; was used for plasmid generation |  | This study |
| *E*. *coli* strain DH5α (pMK2) | Lab strain harbouring the pMK2 plasmid; was used for plasmid generation |  | This study |
| *E*. *coli* strain DH5α (pLS1) | Lab strain harbouring the pLS1 plasmid; was used for plasmid generation |  | This study |
| *E*. *coli* strain DH5α (pLS2) | Lab strain harbouring the pLS2 plasmid; was used for plasmid generation |  | This study |
| *E*. *coli* 83972 | Asymptomatic bacteriuria strain |  | ([Lindberg et al., 1975](#_ENREF_20)) |
| *E*. *coli* 83972 (pKD46) | *E*. *coli* 83972; harbouring the plasmid pKD46 for Red/ET recombineering ([Datsenko and Wanner, 2000](#_ENREF_12)) |  | This study |
| *E*. *coli* 83972 ∆*luxS* | *E*. *coli* 83972 AI-2 synthase *luxS* mutant strain; *luxS*::*Sh ble* |  | This study |
| *E*. *coli* 83972 ∆*ybhC* | *E*. *coli* 83972 *ybhC* mutant strain; *ybhC*::*cat* |  | This study |
| *E*. *coli* 83972 ∆*luxS* (pKD46) | *E*. *coli* 83972 ∆*luxS*; harbouring the plasmid pKD46 for Red/ET recombineering ([Datsenko and Wanner, 2000](#_ENREF_12)) |  | This study |
| *E*. *coli* 83972 *attB*::*lsr* | *E*. *coli* 83972; full-length *lsr* locus with flanking resistance genes (*cat*-*lsrRK*-*lsrACDBFG*-*aph*) integrated into the λ phage *attB* integration site (reviewed in ([Campbell, 1992](#_ENREF_9))) |  | This study |
| *E*. *coli* 83972 ∆*luxS* *attB*::*lsr* | *E*. *coli* 83972 ∆*luxS*; full-length *lsr* locus with flanking resistance genes (*cat*-*lsrRK*-*lsrACDBFG*-*aph*) integrated into the λ phage *attB* integration site (reviewed in ([Campbell, 1992](#_ENREF_9))) |  | This study |
| *E*. *coli* 83972 (pLS2) | *E*. *coli* 83972; harbouring the plasmid pLS2 |  | This study |
| *E*. *coli* 83972 *attB*::*lsr* (pLS1) | *E*. *coli* 83972 *attB*::*lsr*; harbouring the plasmid pLS1 |  | This study |

**Supplementary Table 6: Primers used in this study.**

| Oligonucleotides | Sequence (5’ 🡪 3’) |
| --- | --- |
| bla-TEM-r | ACGCTCACCGGCTCCAGATTTAT |
| CBL_fwd | ATACTCAAAAACTAACAGCCACGGTCATCATGATGTGGCTGTCAATGAAAGCGCCGAATAAATACCTGTG |
| CBL_rev | TGGTTGATCATGGACTGACGACGTCGTTATGGAAAGCGCCTGGGTTATAGTGCCATTCATCCGCTTATTA |
| CFT073_∆luxS_CP_fwd | TGATACTGGTATTGGCGGAA |
| CFT073_∆luxS_CP_rev | CAATGGAAGCAGCAAAATGC |
| CFT073_lsrRK_YFP_CP1_fwd | TAATCACTCTGCCAGATGGC |
| CFT073_lsrRK_YFP_CP2_rev | CGGTTTGATCAGAAGGACG |
| del_ybhC_fwd | TCCCGTTTCGCTCAAGTTAGTATAAAAAAGCAGGCTTCAACGGATTCATTCAAGGCGATTAAGTTGGGTA |
| del_ybhC_rev | GTAGGCCGGATAAGGCGTTTACGCCGCATCCGGCACATAGTTAACAGCTCCCCCAGGCTTTACACTTTAT |
| LKRS_fwd | CAAAAACTAACAGCCACGGT |
| LZP18 | GGGCAAGAATGTGAATAAAG |
| LZP50 | TCGTAGTAAGCATGAACGCC |
| MC_185 | AGGAAACAGCTATGACCATGATTAC |
| pKD3_lsr_seq_12 | GTGGGCAAAGATATGGTGC |
| pKD3_lsr_seq_13 | GCAGGGGGAGAAAATAAAGC |
| pKD3_lsr_seq_4 | TCCAGGTCGAATATCACAGC |
| pMB54_lsrRK_CP1_rev | ATGCGGCGATTGTATCAGC |
| pWKS30_attB_fwd | AATGAATCCGTTGAAGCCTGCTTTTTTATACTAACTTGAGCGAAACGGGACAAGGCGATTAAGTTGGGTA |
| pWKS30_attB_rev | CATCTGGTATCACTTAAAGGTATTAAAAACAACTTTTTGTCTTTTTACCTCCCCAGGCTTTACACTTTAT |
| RBL_fwd | TTAATGACCGGGCCGCGTAAAAAACGTCTGTTCAATGGTTTGATGCCGTGATGGACAACAAGCCAGGGAT |
| RBL_rev | CTCCCGTCAGCGCGTTACCCCAATTGTAGATAAAATTGATAAATTCGCCGCCAGGCATCAAATTAAGCAG |
| Rec_BleoR_rev | CGGGCGGTGGTGTCACCTGCCCGGCTTCTTTTGTTCCACTGATCAGTCCTGCTCCTCGGCCACGAAGTGC |
| Rec_BleoR_fwd | GAACCGTTTGCAGTGTGGCTGGAAAAACACGCCTGACAGAAATGTTGACAATTAATCATCGGCATAGTAT |
| wt_PybhC | CTGCGATGTTTAGTTAATCAC |

**Supplementary Table 7: Plasmids used in this study.**

| Plasmid | Description | Reference |
| --- | --- | --- |
| pKD46 | Encodes the lambda Red genes (*exo*, *bet*, *gam*) for Red/ET recombineering  oriR101 w/repA101ts | ([Datsenko and Wanner, 2000](#_ENREF_12)) |
| pMB54 | *cat* template plasmid | ([Berger et al., 2016](#_ENREF_7)) |
| pKD4 | *aph* template plasmid | ([Datsenko and Wanner, 2000](#_ENREF_12)) |
| pEM7/Zeo | *Sh ble* template plasmid  pBR322 ori | Invitrogen |
| pWKS30 | Low-copy number plasmid  pSC101 ori | ([Wang and Kushner, 1991](#_ENREF_32)) |
| pWKS30_LSR | Template for full-length *lsr* locus complementation | This study |
| pMK1 | Low-copy number plasmid harbouring the *lsrRK*:*lsrA*-promoter-*yfp* fusion  pSC101 ori | ([Keizers et al., 2022](#_ENREF_17)) |
| pMK2 | Low-copy number plasmid harbouring the *lsrR*:*lsrA*-promoter:*yfp* fusion  pSC101 ori | This study |
| pPS1 | Template for the P*dps*-*cfp* cassette | ([Schiller et al., 2021](#_ENREF_30)) |
| pPS2 | Template for the P*dps*-y*fp* cassette | ([Schiller et al., 2021](#_ENREF_30)) |
| pLS1 | Low-copy number plasmid pWKS30 harbouring the P*dps*-*cfp* cassette | This study |
| pLS2 | Low-copy number plasmid pWKS30 harbouring the P*dps*-y*fp* cassette | This study |
| pLP2 | *cat* template plasmid | ([Peng et al., 2022](#_ENREF_26)) |

**Methods**

**Biofilm formation analysis**

Biofilm formation analysis was done according to Laganenka *et al*. ([Laganenka et al., 2016](#_ENREF_19)). Briefly, overnight cultures were diluted to a final optical density OD_600_ = 0.05 in tryptic broth (TB; 10 g/L tryptone and 5 g/L NaCl). 300 µL of freshly diluted cultures were added into one well of a 96-well plate (Thermo Fisher Scientific, Schwerte, Germany) in duplicates for each biological replicate. The plate was incubated statically at 37 °C for 24 h. After incubation, the cultures’ optical densities of 595 nm (± 10 nm) were measured in an Infinite F200 plate reader (TECAN, Männedorf, Switzerland). The wells were rinsed twice with ddH_2_O, and 300 µL of 0.5 % crystal violet solution was added to each well. After incubation at room temperature for 15 min, the wells were rinsed twice with ddH_2_O. The remaining crystal violet was resuspended in 300 µL 96 % ethanol per well, and the optical density (595nm ± 10 nm) was measured. Normalised biofilm formation was calculated by dividing the crystal violet optical density by the culture optical density.

**Supplementary Figures**

**
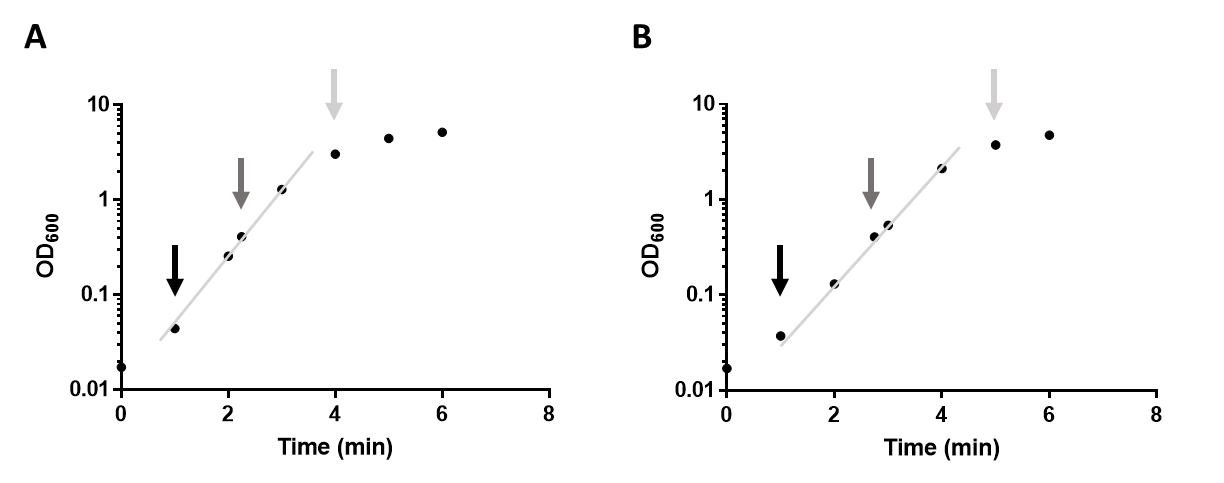
Supplementary Figure 1: Growth curves of the bacterial cultures used for RNA extraction.** Overnight cultures of **(A)** *E*. *coli* 83972 and **(B)** *E*. *coli* 83972 *attB*::*lsr* were diluted to a final OD_600_ = 0.02 in 200 mL lysogeny broth and grown at 37 °C and 180 rpm. Optical densities were measured at the indicated time points. The grey line indicates logarithmic growth. To isolate total RNA, samples were taken at the time points indicated by arrows (black – lag phase, dark grey – exp. phase, light grey – stat. phase). Depicted is the mean of three biological replicates (error bars are too small to be depicted).

**
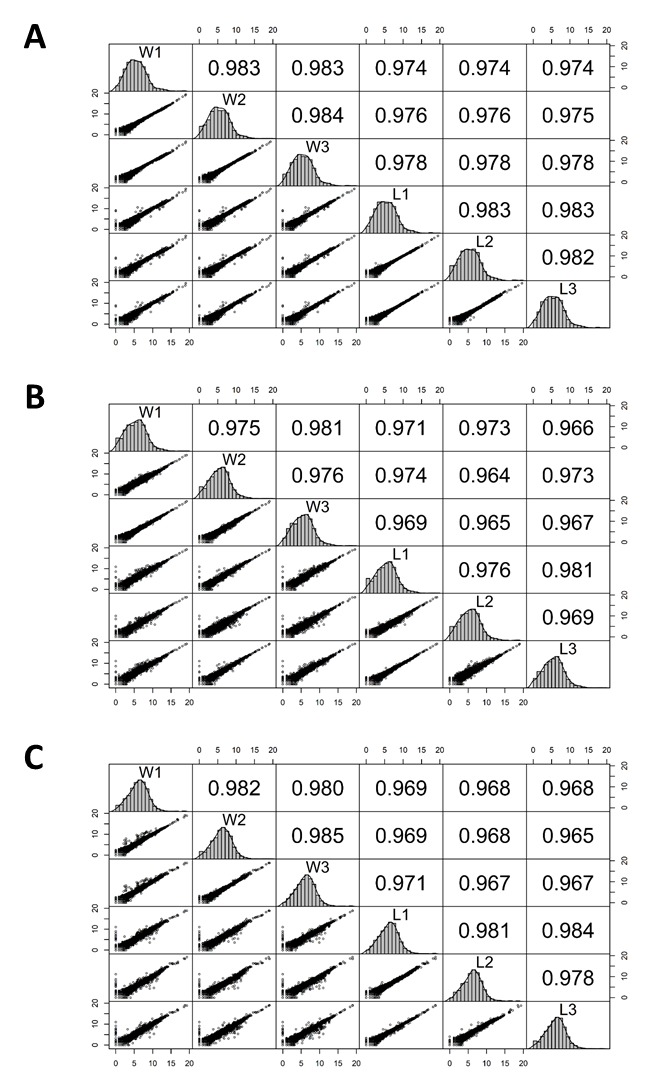
**

**Supplementary Figure 2: Scatterplots of differentially expressed genes in *E*. *coli* 83972 *attB*::*lsr* compared to wild type *E*. *coli* 83972.** Differentially expressed genes (DEG) were computed by analysing the RNAseq data with DESeq2 ([Love et al., 2014](#_ENREF_22)). The counts were normalised, and independent filtering was not used. All analysis was done in R v4.2.2 (R). The scatterplot was computed using pairs.panels**()** from the package psych ([Revelle, 2023](#_ENREF_27)). Other packages used were MASS ([Ripley et al., 2023](#_ENREF_28)), rio ([Becker et al., 2021](#_ENREF_5)), magrittr ([Bache et al., 2022](#_ENREF_3)) and ggplot2 ([Wickham et al., 2023](#_ENREF_33)). Shown are the scatterplots (bottom), the histograms (middle row) and the correlation coefficients (top) between the three biological replicates of *E*. *coli* 83972 (W1-W3) and *E*. *coli* 83972 *attB*::*lsr* (L1-L3) **(A)** in the lag phase, **(B)** during mid-exponential growth and **(C)** during the transition to the stationary phase.


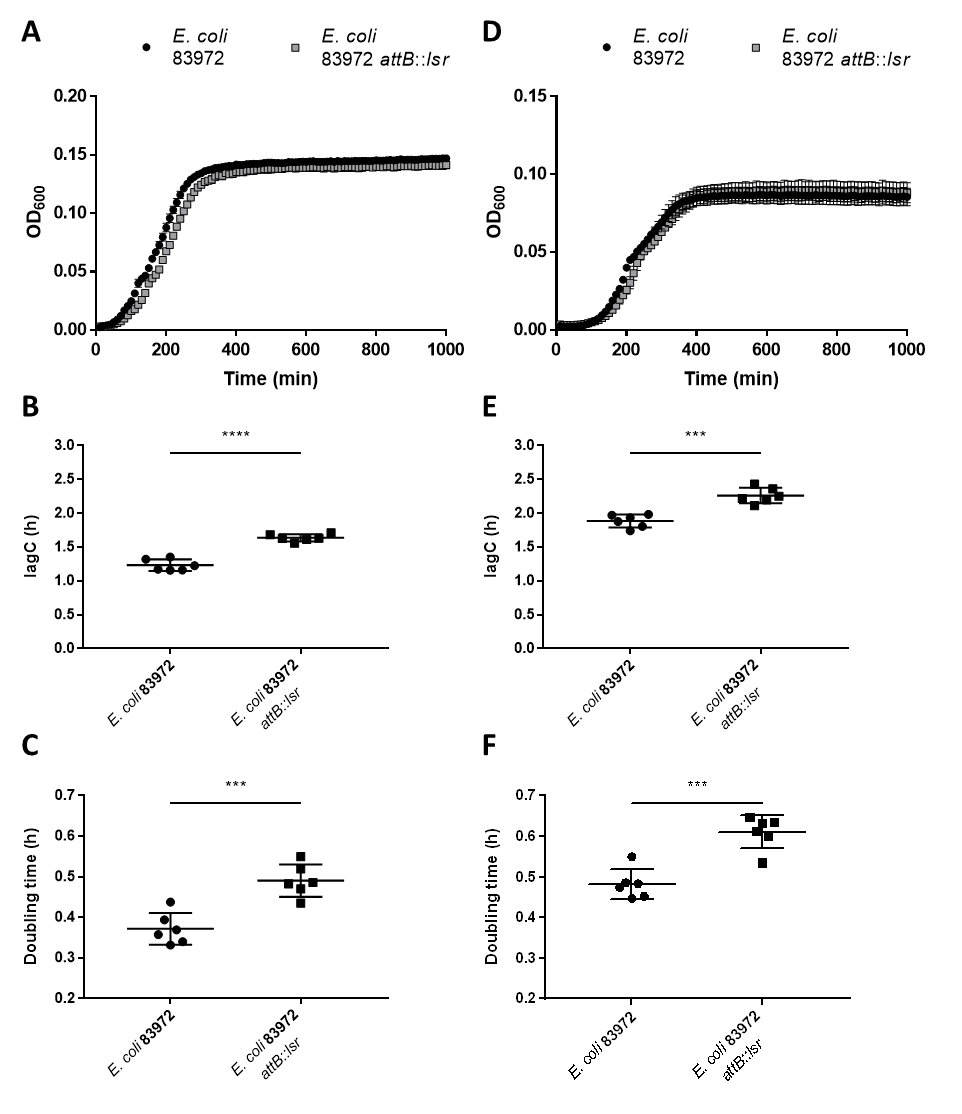


**Supplementary Figure 3: Reintroduction of the *lsr* locus impairs growth of *E*. *coli* 83972 in pooled human urine.** Growth analysis was done in pooled human urine under **(A-C)** aerobic and **(D-F)** anaerobic conditions. **(A & D)** Growth curves over a time span of 1000 min with optical density (OD_600_) measurements every 10 min of *E*. *coli* 83972 (black circles) and *E*. *coli* 83972 *attB::lsr* (grey squares). **(B & E)** Time until cultures reach the exponential growth phase (lagC). **(C & F)** Doubling time during the exponential growth phase. Depicted are the results of three biological replicates in duplicates each. The starting OD_600_ was 0.01 for both strains. Growth curve analysis was done using AMiGA ([Midani et al., 2021](#_ENREF_24)). Statistical analysis was performed using unpaired t-tests; values < 0.05 were considered statistically significant.


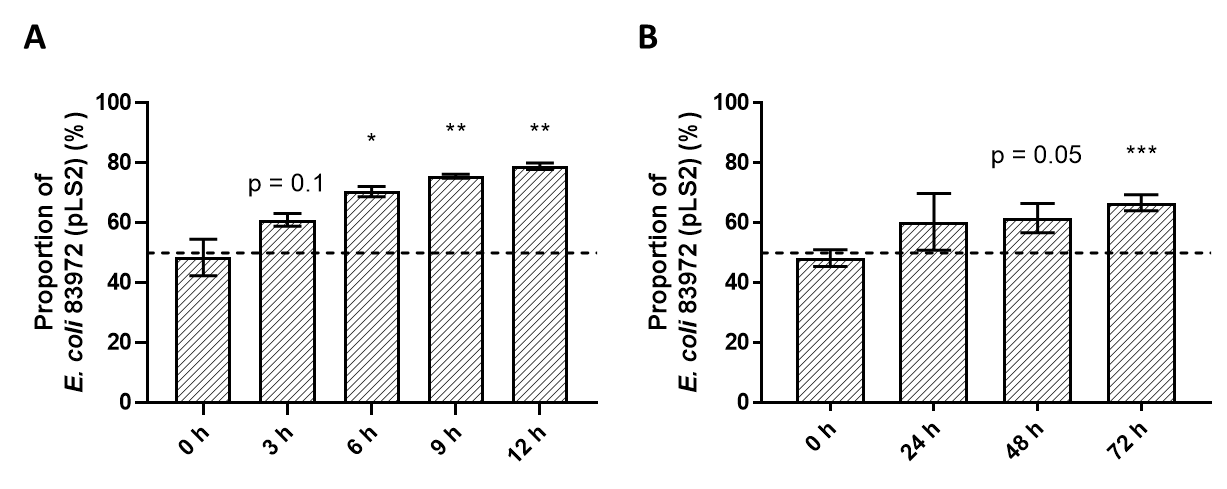


**Supplementary Figure 4: *E*. *coli* 83972 (pLS2) overgrows *E*. *coli* 83972 *attB*::*lsr* (pLS1) in direct competition in pooled human urine.** Competitions were done by mixing overnight cultures of *E*. *coli* 83972 and *E*. *coli* 83972 *attB*::*lsr* in a 1:1 ratio in pooled human urine. **(A)** The cultures were grown with subsequent dilution (1:10) into fresh medium every 3 h. Depicted are the means and standard deviations of the ratio analysis of ten microscopic pictures for four biological replicates at the indicated time points. **(B)** The cultures were grown over a total of 72 hours. Depicted are the means and standard deviations of the ratio analysis of ten microscopic pictures for three biological replicates at the indicated time points. Statistical analysis was performed using RM one-way ANOVA (Geisser-Greenhouse correction) with Dunnett’s multiple comparison test; values < 0.05 were considered statistically significant. *E*. *coli* 83972 (pLS2) overgrew *E*. *coli* 83972 *attB*::*lsr* (pLS1) after one subdilution and overgrew *E*. *coli* 83972 *attB*::*lsr* (pLS1) more after every subsequent subdilution. Without additional subdilutions, *E*. *coli* 83972 (pLS2) has overgrown *E*. *coli* 83972 *attB*::*lsr* (pLS1) after 72 h.

**Supplementary Figure 5: The doubling time of *E*. *coli* strains 83972, 83972 ∆*ybhC*, and 83972 *attB*::*lsr* is not influenced by the initial addition of H_2_O_2_.** Shown is the doubling time during the growth of the *E*. *coli* strains 83972, 83972 ∆*ybhC* and 83972 *attB*::*lsr* in lysogeny broth. Doubling time was analysed using AMiGA ([Midani et al., 2021](#_ENREF_24)). Statistical analysis was performed using ordinary two-way ANOVA with Tukey‘s multiple comparison test. The comparison was made within each family (*E*. *coli* 83972, *E*. *coli* 83972 ∆*ybhC* and *E*. *coli* 83972 *attB*::*lsr*; simple effect within rows); values < 0.05 were considered statistically significant. The doubling time of *E*. *coli* 83972 *attB*::*lsr* was longer than for *E*. *coli* 83972 and *E*. *coli* 83972 *∆ybhC*. However, within each genetic background, there was no statistically significant difference in the doubling time when the bacteria reached the logarithmic growth phase after the H_2_O_2_ challenge. Error bars represent the results of three biological replicates.


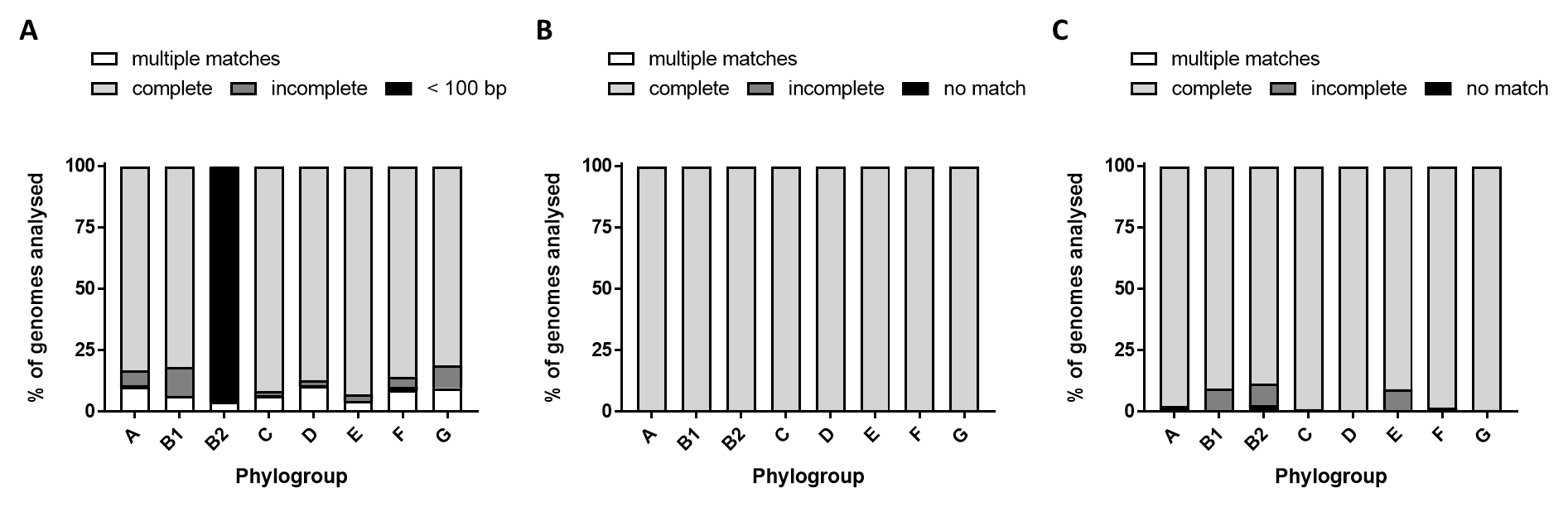


**Supplementary Figure 6: The *lsr* locus is absent in the majority of *E*. *coli* strains of the phylogroup B2, while the presence of the *luxS* and *tam* genes is conserved in *E*. *coli* strains independent of their phylogroup.** Shown is the percentage of genomes which are positive for **(A)** the *lsr* locus, **(B)** the *luxS* gene and **(C)** the *tam* gene in the respective phylogroups of 32,404 *E*. *coli* genomes. The phylogroup was determined using ClermonTyping ([Beghain et al., 2018](#_ENREF_6)), and gene conservation was determined using local BLAST+ ([Camacho et al., 2009](#_ENREF_8)). “Complete” equals a blastn match of **(A)** 8,673 bp, **(B)** 516 bp or **(C)** 759 bp, “incomplete” equals a blastn match **(A)** < 8673 bp, **(B)** < 516 bp or **(C)** < 759 bp, “< 100 bp” equals a blastn match length shorter than 100 bp, “no match” equals no blastn match and “multiple matches” equals multiple blastn matches. While *luxS* and *tam* were detected in > 88 % of all analysed *E*. *coli* strains independent of their phylogroup, > 95 % of *E*. *coli* of the phylogroup B2 only had a homologous region of the *lsr* locus that is shorter than 100 bp. Conversely, > 81 % of *E*. *coli* strains belonging to the phylogroups A, B1, C, D, E, F, and G carried a full-length *lsr* locus.

**Supplementary Figure 7: Biofilm formation analysis of *E*. *coli* strains 83972 and 83972 *attB*::*lsr* in TB.** Depicted is the normalised biofilm formation of *E*. *coli* strains 83972 and 83972 *attB*::*lsr* in TB after 24 h of static growth. Error bars represent the results of three biological replicates in duplicates each. The starting OD_600_ was 0.05 for both strains. Statistical analysis was performed using an unpaired t-test; a value < 0.05 was considered statistically significant.


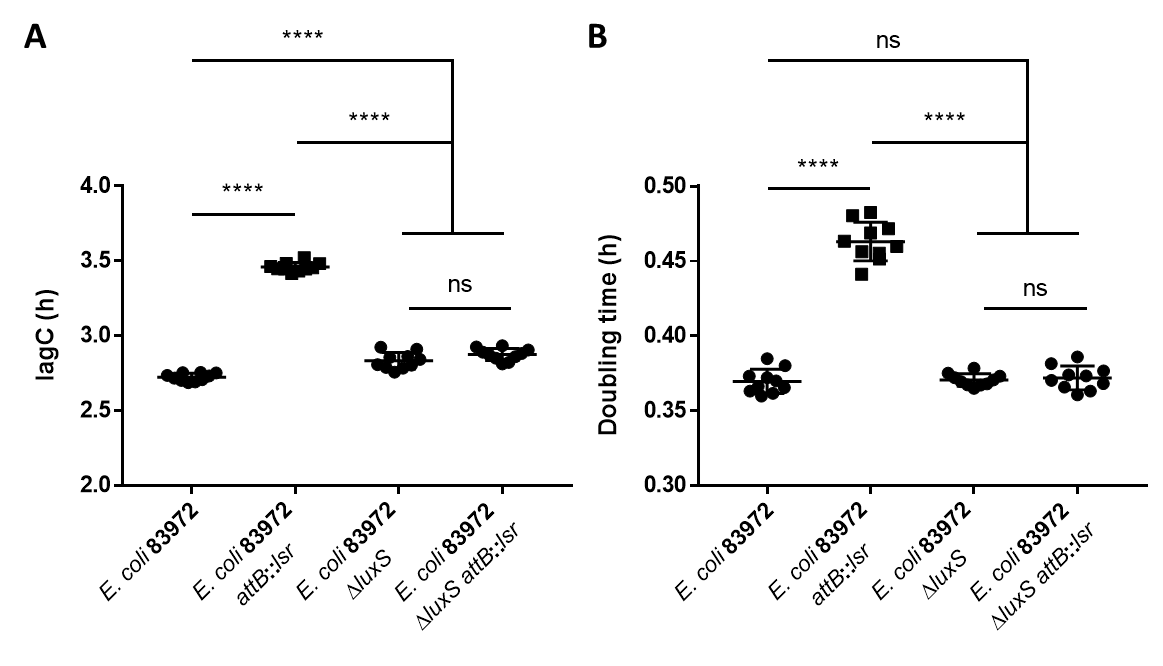


**Supplementary Figure 8: Introduction of the *lsr* locus and deletion of *luxS* lead to significantly longer lag phases as compared to the wild type, while the doubling time is only affected by *lsr* locus complementation.** Growth analysis was done under aerobic conditions in lysogeny broth. **(A)** Time until cultures reach the exponential growth phase (lagC). Statistical analysis was performed using ordinary one-way ANOVA with Tukey‘s multiple comparison test; values < 0.05 were considered statistically significant. **(B)** Doubling time during the exponential growth phase. Statistical analysis was performed using Brown-Forsythe and Welch ANOVA with Holm-Sidak‘s multiple comparison test; values < 0.05 were considered statistically significant. Depicted are the results of five biological replicates in duplicates each. Growth curve analysis was done using AMiGA ([Midani et al., 2021](#_ENREF_24)). *E*. *coli* 83972 *attB*::*lsr* showed a significantly longer lag phase and significantly higher doubling time than *E*. *coli* 83972. The deletion of *luxS* in *E*. *coli* 83972 or *E*. *coli* 83972 *attB*::*lsr* also led to significantly longer lag phases compared to *E*. *coli* 83972, while the lag phases were significantly shorter than *E*. *coli* 83972 *attB*::*lsr*. The doubling time was not affected by a *luxS* deletion, independent of the presence of a full-length *lsr* locus.

**References**

Abdelwahed, E.K., Hussein, N.A., Moustafa, A., Moneib, N.A., and Aziz, R.K. (2022). Gene Networks and Pathways Involved in *Escherichia coli* Response to Multiple Stressors. *Microorganisms* 10, 1793.

Bache, S.M., Wickham, H., and Henry, L. (2022). Package ‘magrittr’: A Forward-Pipe Operator for R. Available: https://cloud.r-project.org/web/packages/magrittr/magrittr.pdf.

Bachmann, B.J. (1972). Pedigrees of some mutant strains of *Escherichia coli* K-12. *Bacteriol Rev* 36, 525-557.

Becker, J., Chan, C.H., Schoch, D., Chan, G.C.H., Leeper, T.J., Gandrud, C., Macdonald, A., Zahn, I., Stadlmann, S., Williamson, R., Kennedy, P., Price, R., Davis, T.L., Day, N., Denney, B., Bokov, A., and Gruson, H. (2021). rio: A Swiss-Army Knife for Data I/O. Available: https://cran.r-project.org/web/packages/rio/index.html.

Beghain, J., Bridier-Nahmias, A., Le Nagard, H., Denamur, E., and Clermont, O. (2018). ClermonTyping: an easy-to-use and accurate in silico method for *Escherichia* genus strain phylotyping. *Microb Genom* 4, e000192.

Berger, M., Gerganova, V., Berger, P., Rapiteanu, R., Lisicovas, V., and Dobrindt, U. (2016). Genes on a Wire: The Nucleoid-Associated Protein HU Insulates Transcription Units in *Escherichia coli*. *Sci Rep* 6, 31512.

Camacho, C., Coulouris, G., Avagyan, V., Ma, N., Papadopoulos, J., Bealer, K., and Madden, T.L. (2009). BLAST+: architecture and applications. *BMC Bioinformatics* 10, 421.

Campbell, A.M. (1992). Chromosomal insertion sites for phages and plasmids. *J Bacteriol* 174, 7495-7499.

Chen, H., Wilson, J., Ercanbrack, C., Smith, H., Gan, Q., and Fan, C. (2021). Genome-Wide Screening of Oxidizing Agent Resistance Genes in *Escherichia coli*. *Antioxidants* 10, 861.

Chiang, S.M., and Schellhorn, H.E. (2012). Regulators of oxidative stress response genes in *Escherichia coli* and their functional conservation in bacteria. *Arch Biochem Biophys* 525, 161-169.

Datsenko, K.A., and Wanner, B.L. (2000). One-step inactivation of chromosomal genes in *Escherichia coli* K-12 using PCR products. *Proc Natl Acad Sci USA* 97, 6640-6645.

Gao, Q., Xia, L., Wang, X., Ye, Z., Liu, J., and Gao, S. (2019). SodA Contributes to the Virulence of Avian Pathogenic *Escherichia coli* O2 Strain E058 in Experimentally Infected Chickens. *J Bacteriol* 201, e00625-18.

Kanehisa, M. (2019). Toward understanding the origin and evolution of cellular organisms. *Protein Sci* 28, 1947-1951.

Kanehisa, M., Furumichi, M., Sato, Y., Kawashima, M., and Ishiguro-Watanabe, M. (2023). KEGG for taxonomy-based analysis of pathways and genomes. *Nucleic Acids Res* 51, D587-D592.

Kanehisa, M., and Goto, S. (2000). KEGG: Kyoto Encyclopedia of Genes and Genomes. *Nucleic Acids Res* 28, 27-30.

Keizers, M., Dobrindt, U., and Berger, M. (2022). A Simple Biosensor-Based Assay for Quantitative Autoinducer-2 Analysis. *ACS Synth Biol* 11, 747-759.

Kitts, P.A., Church, D.M., Thibaud-Nissen, F., Choi, J., Hem, V., Sapojnikov, V., Smith, R.G., Tatusova, T., Xiang, C., Zherikov, A., Dicuccio, M., Murphy, T.D., Pruitt, K.D., and Kimchi, A. (2016). Assembly: a resource for assembled genomes at NCBI. *Nucleic Acids Res* 44, D73-80.

Laganenka, L., Colin, R., and Sourjik, V. (2016). Chemotaxis towards autoinducer 2 mediates autoaggregation in *Escherichia coli*. *Nat Commun* 7, 12984.

Lindberg, U., Hanson, L.A., Jodal, U., Lidin-Janson, G., Lincoln, K., and Olling, S. (1975). Asymptomatic bacteriuria in schoolgirls. II. Differences in *Escherichia coli* causing asymptomatic bacteriuria. *Acta Paediatr Scand* 64, 432-436.

Lopez, C., Checa, S.K., and Soncini, F.C. (2018). CpxR/CpxA Controls *scsABCD* Transcription To Counteract Copper and Oxidative Stress in *Salmonella enterica* Serovar Typhimurium. *J Bacteriol* 200, e00126-00118.

Love, M.I., Huber, W., and Anders, S. (2014). Moderated estimation of fold change and dispersion for RNA-seq data with DESeq2. *Genome Biol* 15, 550.

Manchado, M., Michan, C., and Pueyo, C. (2000). Hydrogen peroxide activates the SoxRS regulon *in vivo*. *J Bacteriol* 182, 6842-6844.

Midani, F.S., Collins, J., and Britton, R.A. (2021). AMiGA: Software for Automated Analysis of Microbial Growth Assays. *mSystems* 6, e0050821.

Monje-Casas, F., Jurado, J., Prieto-Alamo, M.J., Holmgren, A., and Pueyo, C. (2001). Expression analysis of the *nrdHIEF* operon from *Escherichia coli*. Conditions that trigger the transcript level *in vivo*. *J Biol Chem* 276, 18031-18037.

Peng, L., Dumevi, R.M., Chitto, M., Haarmann, N., Berger, P., Koudelka, G., Schmidt, H., Mellmann, A., Dobrindt, U., and Berger, M. (2022). A Robust One-Step Recombineering System for Enterohemorrhagic *Escherichia coli*. *Microorganisms* 10, 1689.

*R: The R Project for Statistical Computing* [Online]. Available: https://[www.r-project.org/](http://www.r-project.org/) .

Revelle, W. (2023). Package ‘psych’: Procedures for Psychological, Psychometric, and Personality Research. Available: https://cran.r-project.org/web/packages/psych/psych.pdf.

Ripley, B., Venables, B., Bates, D.M., Hornik, K., Gebhardt, A., and Firth, D. (2023). Package ‘MASS’: Support Functions and Datasets for Venables and Ripley’s MASS. Available: https://cran.r-project.org/web/packages/MASS/MASS.pdf.

Roth, M., Jaquet, V., Lemeille, S., Bonetti, E.J., Cambet, Y., Francois, P., and Krause, K.H. (2022). Transcriptomic Analysis of *E. coli* after Exposure to a Sublethal Concentration of Hydrogen Peroxide Revealed a Coordinated Up-Regulation of the Cysteine Biosynthesis Pathway. *Antioxidants* 11, 655.

Schiller, P., Knödler, M., Berger, P., Greune, L., Fruth, A., Mellmann, A., Dersch, P., Berger, M., and Dobrindt, U. (2021). The Superior Adherence Phenotype of *E. coli* O104:H4 is Directly Mediated by the Aggregative Adherence Fimbriae Type I. *Virulence* 12, 346-359.

Seaver, L.C., and Imlay, J.A. (2001). Alkyl hydroperoxide reductase is the primary scavenger of endogenous hydrogen peroxide in *Escherichia coli*. *J Bacteriol* 183, 7173-7181.

Wang, R.F., and Kushner, S.R. (1991). Construction of versatile low-copy-number vectors for cloning, sequencing and gene expression in *Escherichia coli*. *Gene* 100, 195-199.

Wickham, H., Chang, C., Henry, L., Lin Pedersen, T., Takahashi, K., Wilke, C., Woo, K., Yutani, H., Dunnington, D., and Van Den Brand, T. (2023). *Package ‘ggplot2’: Create Elegant Data Visualisations Using the Grammar of Graphics*. Available: https://cran.r-project.org/web/packages/ggplot2/ggplot2.pdf.

Zheng, M., Wang, X., Templeton, L.J., Smulski, D.R., Larossa, R.A., and Storz, G. (2001). DNA microarray-mediated transcriptional profiling of the *Escherichia coli* response to hydrogen peroxide. *J Bacteriol* 183, 4562-4570.
